# The impact of poverty reduction and development interventions on non-communicable diseases and their behavioural risk factors in low and lower-middle income countries: A systematic review

**DOI:** 10.1371/journal.pone.0193378

**Published:** 2018-02-23

**Authors:** Jessie Pullar, Luke Allen, Nick Townsend, Julianne Williams, Charlie Foster, Nia Roberts, Mike Rayner, Bente Mikkelsen, Francesco Branca, Kremlin Wickramasinghe

**Affiliations:** 1 British Heart Foundation Centre on Population Approaches for NCD Prevention, Nuffield Department of Population Health, University of Oxford, Oxford, United Kingdom; 2 Health Library, Nuffield Department of Population Health, University of Oxford, Oxford, United Kingdom; 3 WHO Global Coordination Mechanism on Non-Communicable Diseases, WHO Headquarters, Geneva, Switzerland; Universiteit Gent, BELGIUM

## Abstract

**Introduction:**

Non-communicable diseases (NCDs) disproportionately affect low- and lower-middle income countries (LLMICs) where 80% of global NCD related deaths occur. LLMICs are the primary focus of interventions to address development and poverty indicators. We aimed to synthesise the evidence of these interventions' impact on the four primary NCDs (cardiovascular disease, diabetes, chronic respiratory disease and cancer) and their common behavioural risk factors (unhealthy diets, physical inactivity, tobacco and alcohol use).

**Methods:**

We systematically searched four online databases (Medline, Embase, Web of Science and Global Health) for primary research conducted in LLMICS, published between January 1st 1990 and February 15th 2016. Studies involved development or poverty interventions which reported on outcomes relating to NCDs. We extracted summary level data on study design, population, health outcomes and potential confounders.

**Results:**

From 6383 search results, 29 studies from 24 LLMICs published between 1999 and 2015 met our inclusion criteria. The quality of included studies was limited and heterogeneity of outcome measures required narrative synthesis. One study measured impact on NCD prevalence, one physical activity and 27 dietary components. The majority of papers (23), involved agricultural interventions. Primary outcome measures tended to focus on undernutrition. Intensive agricultural interventions were associated with improved calorie, vitamin, fruit and vegetable intake. However, positive impacts were reliant on participant's land ownership, infection status and limited in generalisability. Just three studies measured adult obesity; two indicated increased income and consequential food affordability had the potential to increase obesity. Overall, there was poor alignment between included studies outcome measures and the key policy options and objectives of the Global Action Plan on NCDs.

**Conclusions:**

Though many interventions addressing poverty and development have great potential to impact on NCD prevalence and risk, most fail to measure or report these outcomes. Current evidence is limited to behavioural risk factors, namely diet and suggests a positive impact of agricultural-based food security programmes on dietary indicators. However, studies investigating the impact of improved income on obesity tend to show an increased risk. Embedding NCD impact evaluation into development programmes is crucial in the context of the Sustainable Development Goals and the rapid epidemiological transitions facing LLMICs.

## Introduction

Non-communicable diseases (NCDs) predominate the global disease burden, disproportionally affecting low and lower-middle income countries (LLMICs)[1]. Latest estimates suggest 82% of premature deaths attributed to NCDs occur in LLMICs, with further increases predicted in the prevalence of the four most prevalent NCDs- cardiovascular disease (CVD), type 2 diabetes (T2DM), cancer and chronic obstructive (COPD) respiratory diseases [1]. This growing burden compounds already stressed health systems, disproportionately affecting the poorest populations and hindering countries social and economic development [2].

Since the late 1990s, the Millennium Development Goals (MDGs) have formed the basis of work in health, poverty and development within LLMICs [3]. Reflecting the key issues when they commenced, the MDGs have focused development initiatives towards supporting poverty reduction, education, gender equality, environmental sustainability, child mortality, maternal health and the control of infectious diseases. While great progress in these areas has been made, globalization and resulting epidemiological and nutrition transitions has led to the rapid emergence of NCDs within LLMICs [4].

Ten years after the inception of MDGs, the global burden of disease study identified major shifts in the magnitude of health risks within LLMICs [1]. Globally, traditional causes of ill health in children (diarrhea, infections, undernutrition) slipped down in risk ratings as NCD related risk factors rose. Though as the burden of NCDs climbs, many LLMICs continue to battle infectious disease epidemics and undernutrition [5]. For example, in Sub-Saharan Africa where malnutrition remains the leading health risk factors, the greatest rise in NCDs has been evidenced. By 2030 46% of deaths within this region are expected to be related to NCDs [6].

The mounting development challenge of NCDs was recognised by the World Health Organisation (WHO), who declared NCDs were ‘putting a break on development, undermining the MDGs and amplifying social inequality’ [7]. The 2011 Political Declaration on the prevention and control of NCDs (Paragraph 1- Resolution A/RES/66/2), concluded the way to combat NCDs was clear–by tackling the four primary modifiable behavioural risk factors for NCDs- specifically, tobacco use, physical inactivity, unhealthy diets and the harmful use of alcohol within all member states, and control of the four diseases [7].

To address these risk factors, the WHOs Global Action Plan (NCD GAP) on NCDs provides a roadmap for governments to address NCDs by outlining key policy options to attain 9 voluntary targets by 2025 [8]. In achieving these targets a 25% reduction in premature mortality from NCDs is predicted- this is particularly important for LLMICs where 82% of premature NCD related deaths occur [1,8]. The first objective of the NCD GAP explicitly outlines the aim of increasing the priority of addressing NCDs within international development agendas through strengthened co-operation and advocacy [8]. This is supported by NCDs inclusion in the Sustainable Development Goals (SDG) [8]. These goals recognize NCDs as a major barrier to sustainable development and aim to achieve a reduction in NCD-related premature mortality by one-third by 2030. The SDGs complement policy objectives and interventions of the GAP NCDs to encourage cohesive action by member states and the incorporation of NCDs into national development plans. To support this, a strong evidence base is required to understand ways of effectively linking development agendas to NCD prevention and control in practice.

The promotion of a core set of cost-effective preventative and curative interventions, commonly referred to as ‘Best Buy Interventions’, aims to assist governments in implementing NCD interventions [8–9]. However these are largely focused on health-centric interventions. Recognising many causes and solutions of the NCD burden lie outside of the health system, growing emphasis is placed on supporting multi-sector approaches to NCD prevention. In such approaches, complementary sectors such as agriculture and education work in collaboration with the health sector towards shared goals. Guided by the MDGs and SDGs, many development initiatives have the potential to impact on recipient’s risk of NCDs [5].

To the best of our knowledge, no systematic review has examined the impact of development and poverty reduction interventions on NCDs and behavioural risk factors. Such a review has the potential to provide important evidence about whether or not current development projects are impacting on NCD outcomes and to inform future projects and policy makers catalysing multi-sector approaches for NCD prevention and control. These approaches encourage inter-governmental and inter-agency collaboration to address NCDs through the establishment of synergistic objectives and interventions which can positively impact on areas such as agriculture, education, fisheries management and climate action while also supporting healthy environments for NCD prevention [10].

This systematic review aims to answer the question, ‘what is the evidence for the impact of development interventions on NCD outcomes and behavioural risk factors in LLMICs? Included poverty reduction and development interventions were defined as those that addressed economic development, social inequalities, community engagement, agriculture, fisheries, water or sanitisation or worked towards attaining participant’s human rights. For the purpose of this review we considered the four NCDs which account for 82% of all NCDs. Namely-cardiovascular disease (CVD), type 2 diabetes mellitus (T2DM), cancer and chronic obstructive pulmonary disease (COPD) [8]. We also considered the four common, modifiable risk factors for these diseases- tobacco use, unhealthy diets, physical inactivity and the harmful use of alcohol [8].

This review aims to support ongoing NCD prevention methods and provide evidence for the planning and development of future development and poverty reduction initiatives. This is the third paper in a three-part series examining NCDs in LLMICs.

## Methods

### Protocol and registration

Our review followed PRISMA guidelines for systematic reviews. The supporting PRISMA checklist is available as supplementary information (S1 Table), and PROSPERO registered protocol (PROSPERO: 42016039030, 4201603903). Due to the low number of papers the findings of two PROSPERO protocols (covering NCD outcomes and NCD risk factors) are reported on within this paper. Ethics approval was not required for this work.

### Database and search strategies

We conducted a comprehensive literature search for papers published between January 1^st^ 1990 and February 15^th^ 2016 using a pre- determined search strategy (S2 Table). The search strategy was developed by the medical librarian and MESH terms formulated to cover the PICO elements of the research question. Search terms combined synonyms for poverty and development interventions with terms for NCD morbidity, mortality and behavioural risk factors as well as the 82 LLMIC countries defined according to the World Bank’s 2015 definition [11]. We conducted the search in English on the following electronic databases: MEDLINE, EMBASE, Web of Science Core Collection and Global Health. Additionally, we reviewed the first 30 hits from Google Scholar and searched the websites of WHO, World Bank, United Nations Food and Agricultural Organisation (FAO). Reference lists of key papers identified within the literature search were also reviewed.

### Inclusion criteria

Included studies evaluated the impact of an intervention aimed to address a poverty and/or development indicator on at least one NCD morbidity, mortality or behavioural risk factor outcome measure within LLMICs (S3 Table). The population of interest was development programme recipients within LLMICs, with no restriction placed on age. Definitions of parameters are listed below:

Development and poverty reduction interventions (referred to as development interventions throughout):

- Economic development interventions (including financial management/ support, small business promotion, entrepreneurship, market linkage)
- Social change interventions (including projects which support gender equality, social justice, social support, human rights)
- Social protection interventions (including conditional and unconditional cash transfer, food for work, government subsidies, government taxes, microcredit programmes)
- Employment programmes (training, access, loans)
- Fisheries programmes
- Environmental protection programmes
- Agricultural programmes
- Water and sanitisation programmes
- Infrastructural and facilities improvement programmes

NCD morbidity and mortality outcomes [8]:

- *Cardiovascular disease* -myocardial infarction, heart failure, brain ischemia, stroke, heart disease, coronary artery disease, cerebrovascular events, vascular events, heart failure
- *Diabetes*- type 2 diabetes, non-insulin dependent diabetes, type 1 diabetes, insulin resistance, impaired glucose tolerance
- *Cancer-* neoplasms, carcinoma, tumors, malignancy, leukemia, lymphoma
- *Chronic respiratory diseases*-chronic obstructive pulmonary disease, chronic lung/pulmonary conditions, asthma, lung diseases

NCD behavioural risk factor outcomes [8]:

- *Tobacco use* -tobacco smoking or chewing, tobacco control policies
- *Physical inactivity*—physical activity levels, sedentary behaviour
- *Unhealthy diet-* diet composition, fruit, vegetable, salt, fat, sugar, calorie intake
- *Harmful use of alcohol*- alcohol consumption

Grey literature was excluded. No restrictions were made based on publication language, study design or participant characteristics.

### Study selection and data extraction

Citations from search results were collated in Endnote, duplicates removed, and the final results exported into Excel for eligibility screening. JP and LA independently screened titles and abstracts against the predefined eligibility criteria (S3 Table). To ensure consistency, Cohen’s kappa statistic was calculated at 10% intervals (every 638 papers). Once “excellent agreement” was reached (agreement exceeding 95% and Cohen’s kappa > 0.75) JP screened all remaining records [12]. Uncertainties were brought to LA, KW and NT with disagreements resolved by group consensus. This process was repeated for full text review. For studies where the data was unclear, or more information was required the authors were contacted by email.

Following full text review, references of selected studies were examined for any additional studies of relevance. Data collection was then conducted on all eligible papers. Data collection involved the extraction of key study parameters including country, intervention design, participant population, NCD outcome and quality scoring measures.

### Quality assessment

A quality assessment was conducted on all included papers by JP and LA using validated tools endorsed by Cochrane reviews (S4 Table). For randomised controlled trials (RCTs) the Cochrane Risk of Bias tool was used to evaluate the risk of biased conclusions based on selection, performance, detection, attrition, reporting and other bias [13]. Observational cohorts were assessed using the Newcastle-Ottawa Scale (NOS) [14]. NOS scores studies based on methodological rigor by assessing study selection, comparability of cohorts and outcome assessment. Under each category, studies were assessed based on the coding structure developed by Wells et al [14]. The quality of cross-sectional studies was also assessed using an adapted version of the NOS which assessed selection, comparability and outcome bias using the same letter and star system as used for cohort studies. None of the utlised bias assessment tools have a validated method of summarizing overall bias scores. Instead, the scorings are used to discuss strengths and weaknesses within each paper, as well as common areas of weaknesses at an outcome and review level.

### Synthesis of results

Due to the heterogeneity of included studies design and outcome measures a meta-analysis was not possible. Data was extracted into tables, categorised by NCD outcome and intervention design and a narrative analysis of study results was reported.

## Results

### Study selection

**Fig 1:**
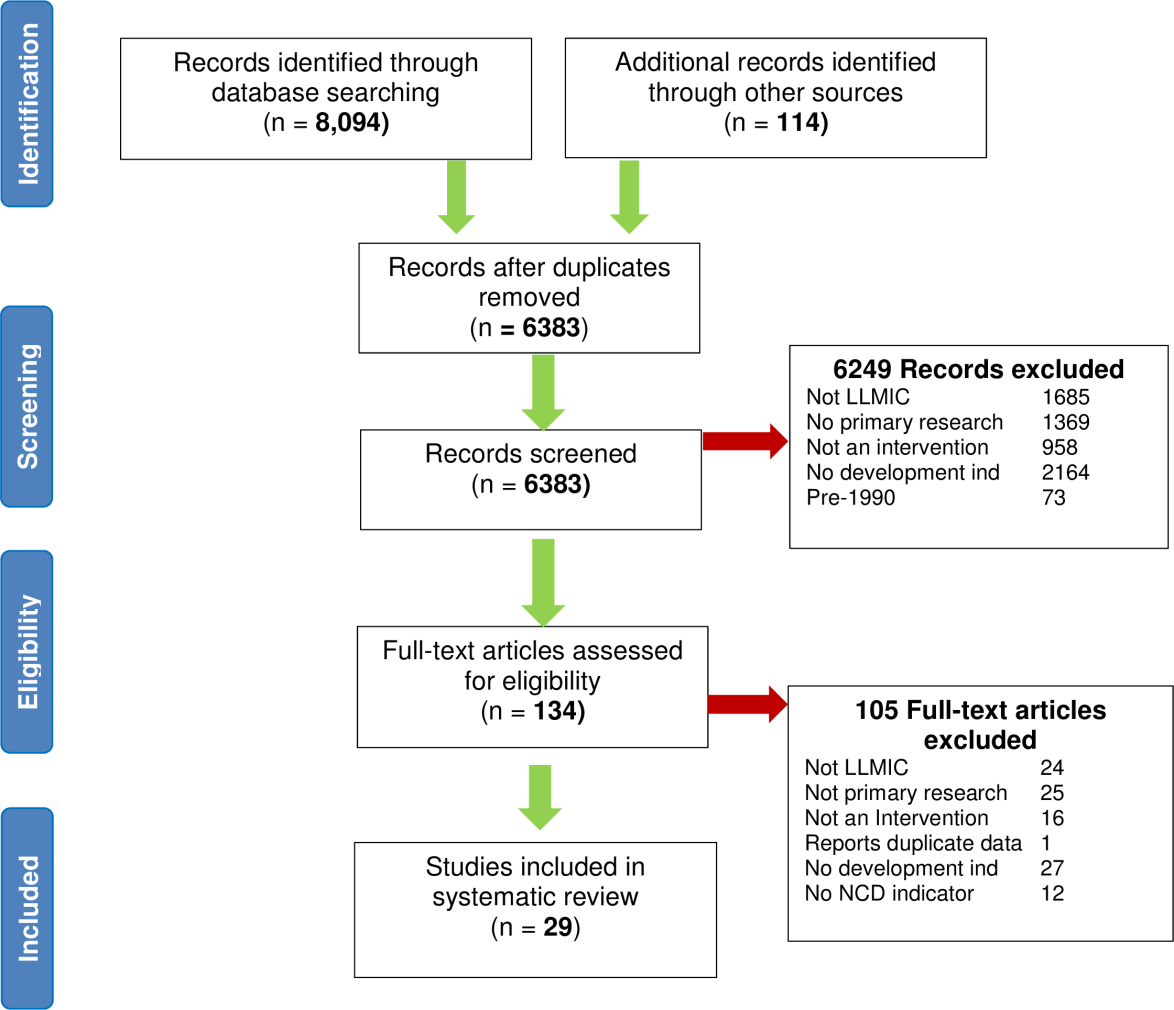
Results of screening process. Number of papers screened and included in review process.

The database search retrieved 8,094 citations with a further 114 identified through additional searches. After the removal of duplicates, 6,383 citations underwent abstract screening, and 134 full text review. Twenty-nine citations met the study inclusion criteria (Fig 1). Primary reasons for exclusion included the absence of development indicator, study not based in an LLMIC, not primary research, not involving an intervention or not including an NCD related outcome measure.

### Study characteristics

**Fig 2:**
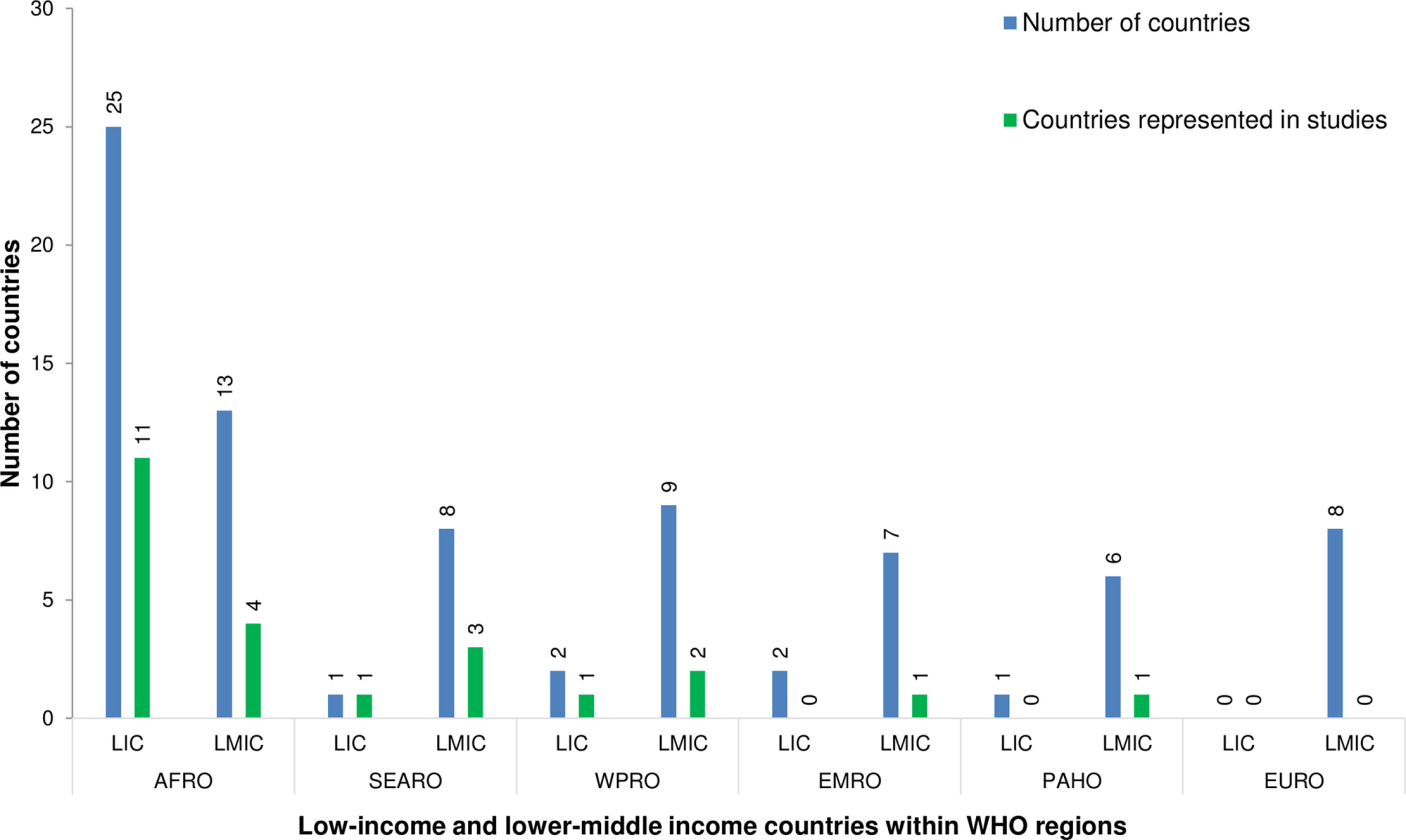
Number of included papers from LICs and LMICs according to WHO region. Number of LIC and LMIC countries within each WHO region, and the number of countries represented by included papers.

Included studies were published between 1999 and 2015, with 66% published after 2010. Studies were conducted in 24 different LLMICs. Of these, 15 were from WHOs African Region (AFRO), nine from South East Asia (SEARO), three from Western Pacific (WPRO), one from Eastern Mediterranean (EMRO) and one from the Americas (PAHO). There were no included studies from the European region (EURO). Fig 2 shows the number of low income (LIC) and lower-middle income (LMIC) countries, along with the number represented by studies within each region.

One study was published in Spanish [15], while 28 were in English. The majority of studies used observational design (20); three modeling and four were randomised controlled trials (RCTs). In relation to participant characteristics, 26 studies were conducted on participants from rural and farming households [15–41], 12 measured outcomes in children [18–23,25,28,33,40–42] and 23 measured adult outcomes (with nine focusing specifically on women[17,19–20,23,26,30,36,42–42]).

Of the included studies 27 reported on dietary outcomes (with 10 including measures of over-nutrition such as calorie consumption, overweight and obesity), one on physical activity and one on diabetes and ischemic heart disease (IHD). The primary development component involved agricultural interventions (23 out of 29 papers), the remaining six involved social protection programmes. Development interventions fell into six broad categories: bio-fortification, social protection programmes, agricultural diversification, livestock and fisheries management, and multi-component interventions.

In all studies measuring dietary factors, the aim of the intervention was to prevent or address undernutrition. Outcome measures of dietary studies varied greatly; the most common measure was diet composition- measured by the intake of different food groups. Three studies used the FAOs Household Food Security and Dietary Diversity Access scale [15,34,39].

Three studies evaluated outcomes at a population level [16,41,43] the remaining 25 measured outcomes at the individual level and were focused on addressing undernutrition, physical drudgery and water contamination.

### Non-communicable diseases

The only study assessing impact on NCD morbidity or mortality to meet inclusion criteria modeled the impact of mitigating arsenic exposure via the substitution of surface water for well water in Bangladesh and the resulting impact on diabetes and ischemic heart disease (IHD) prevalence [16]. Intending to update a previously used model, which suggested a reduction in disability adjusted life years relating to diabetes and IHD through mitigating arsenic exposure. However, investigators determined that further work was required to first establish and quantify the relationship between diabetes, IHD and arsenic exposure [16]. Therefore, no studies reported on the impact of development interventions on NCD morbidity or mortality.

### NCD behavioural risk factors

#### Alcohol and tobacco use

No included studies examined the impact of development interventions on alcohol or tobacco use.

#### Physical inactivity

Only one small observational cohort, involving 120 female hill farm workers in India, measured impact on physical activity (Table 1) [17]. The introduction of an improved clod breaker effectively reduced ‘drudgery’ associated with the farming shown by reduced heart rate and self-perceived exertion. No studies measured the impact of interventions on increasing physical activity or reducing sedentary behaviour.

**Table 1:** Interventions addressing physical activity.

Author, year, country, reference	Study Population (n)	Intervention	Outcome Measures	Outcome
**Kishtwaria J, 2012, India [17]**	Convenience sample of 120 hill farm women from the hill state regions of Himachal Pradesh (HP) and Uttrakhand (UT) (60 from each region) *Control Condition*: Baseline measures of the study population.	The proposed intervention involved the introduction of ergonomic improvement technologies (improved clod breaker) to reduce ergonomic stress during weeding activities tested during 30 minute trial of traditional vs. improved tools.	BMI, VO_2_ max, Heart Rate, energy expenditure	The average heart rate of female hill farmers reduced by 10 beats/min in HP (117–107, p<0.05) and 25 beats/min in UT (125–99, p<0.05) using the improved clod breaker. Average energy expenditure appeared to decline by between 1.6–4 kj/min though the significance of this was not assessed. The average BMI of women was 22.85 HP, 20.17 UT, all women showed high physical fitness (V0_2_ Max 25.35 HP, 32.08 UT ml/min/kg).

#### Unhealthy diets

The remaining 27 studies investigated the impact of interventions on dietary measures [18–43]; these are reported below based on the type of development intervention.

#### Bio-fortification

Four studies examined the impact of bio-fortification focused agricultural interventions on the consumption of vitamin A rich fruits and vegetables (Table 2). Three of these studies involved the promotion of Orange Flesh Sweet Potato (OFSP) [18–20]. All studies identified the promotion and provision of OFSP vines increased the production and consumption of OFSP- consequently increasing vitamin A intake in women and children.

**Table 2:** Bio-fortification interventions impacting on diet.

Study Population (n)	Intervention	Outcome Measures	Outcome
**Low J, 2007, Mozambique [18]**			
733 Children under 5 years recruited from 741 households participating in the program. Households selected by implementing agencies based on proximity, acceptability and vulnerability to malnutrition. Control Condition: Children from matched areas following standard supplementation procedure (only 9.9% received supplements).	Pilot study to establish the efficacy of a food based approach using Orange Flesh Sweet Potato (OFSP) in increasing serum retinol. The two-year intervention involved 3 components- Agricultural (provision of OFSP, agriculture training), demand creation/behaviour change health and nutrition education and activities for women), marketing (training and awareness activities).	Blood samples, nutrition knowledge survey, 24 hour dietary recall, FFQ, anthropometry	In comparison to the control group, household production of OFSP was 79% higher (90% vs. 11%, p <0.001), the percent of children consuming OFSP 3 or more days in the dry season (54% vs. 4%, p<0.001) and wet (55% vs. 8%, p<0.001) was significantly higher following the intervention. Children in the intervention showed significantly higher energy (5921kJ/d (4434–7750) vs. 5133(3839–6699), p<0.001) and vitamin A intake (426 μg RAE/d (61–1902) vs. 56 (24–129), p<0.001) post intervention. Statistically higher intakes of thiamine, riboflavin, Niacin. Vitamin B-6, Folate and Vitamin C were also seen in the intervention group. The mean difference in serum retinol (micromoles per liter), when adjusting for seasonal differences and infection, between groups was 0.076 *μ*mol/L± 0.023, p<0.01. For children in the intervention there was a significant reduction in those diagnosed with VAD (<0.70 μmol/L) over the 2 year trial from 60% to 38%,p<0.01 (controlled for infection) which was significantly greater than in the control group (52% to 53%,p<0.01).
**Hotz C, 2012, Mozambique [19]**			
Measurements from female caretakers (n = 393) and their children aged 6–35 months (n = 386) from a sample of 12 000 households within cluster areas randomised to receive the intervention. Control Group: Matched female caretakers and children from cluster areas not randomised to receive the intervention.	Three-year trial involving the provision of OFSP vines by HarvestPlus to address vitamin A deficiency in women and children. Included training with focus on agricultural, demand creation/ behaviour change and marketing. There were two arms of intervention. Intensive (1) involved more agricultural training, behaviour change and marketing sessions throughout the three-year intervention. In the reduced intervention (2) these sessions ceased after 1 year.	24 hour dietary recall, FFQ, anthropometry to measure OFSP intake and Vitamin A intake.	Vitamin A intake (μg RAE/d ± SE) Impact estimates: Children (1): 202.1 ± 36.3, p<0.01, (2): 206.8 ± 26.1, p<0.01. Women (1): 221.0 ± 96.0, p<0.01, (2): 332.4 ± 89.3,p<0.01. OFSP Intake (g/d) Impact estimates: Children (1): 48.3 ± 12.1, p<0.01, (2): 41.1 ± 13.1, p<0.01. Women (1): 97.4 ± 26.6, p <0.01, (2): 119.4 ± 26.5, p<0.01. After 3 years 26% more households were producing and consuming OFSP. Vitamin A intake was significantly higher in all intervention groups in comparison to controls with OFSP providing between 71–84% of total vitamin A. There were no significant differences between intervention groups. Note baseline data was taken outside of harvest season, while follow up data was taken in peak harvest season.
**Hotz C, 2012, Uganda [20]**			
Children 6–35 months of age (n = 265), children 3–5 y of age (n = 578), and women (n = 573) from rural farming households in one of three study areas. Control Group: Matched households not randomised to receive the intervention: children 3–5 y of age (n = 891), and women (n = 939).	Two-year investigation into the effectiveness of HarvestPlus OFSP on improving vitamin A intake. Involved two armed intervention involving an intensive (1) and reduced (2) program. Both interventions involved three components- Agricultural (provision of 20kg free OFSP vines per household, agriculture training), demand creation/behaviour change (health and nutrition education and activities for women), marketing (training and awareness activities). After year one agricultural components only continued in the intensive program.	24 hour recall, venous blood samples, anthropometry	Net change in OFSP (mean g/day ± SE); 6–35 months (1): 51 ± 13, p<0.01, (2): 37 ± 13, p<0.01. 3–5 years (1): 85 ± 17, p <0.01, (2): 120 ± 29, p<0.01. Women (1): 153 ± 35, p<0.01, (2): 111 ± 26, p <0.001. Significant increases in net OFSP intake in all age groups from baseline. In children aged 12–35 months (>30%) and women (>25%) there was a significant reduction in the prevalence of inadequate vitamin A intakes for both intervention arms (p<0.001).To maximise sensitivity blood serum was only assessed for those with serum retinol <1.05 μmol/L at baseline. In children aged 3–5 years at baseline there was a significant reduction in the percentage with serum retinol <1.05 μmol/L (0.095 ± 0.045, p<0.05) after 2 years. A significant reduction was also seen for children who received deworming (-.006 ± 0.002, p<0.001) and vitamin A supplementation (-0.122 ± 0.056, p<0.05) in the past 12 months. In those same children 79% were at some stage of infection and serum CRP>5.0mg/L was associated with significantly lower serum retinol (-0.222±0.081, p<0.001). No significant difference was seen in the percentage of 3–5 year olds diagnosed with VAD (<0.70 μmol/L), or women’s serum retinol following the intervention

**Kidala D, 2000, Tanzania [21]**			
75 children aged 6–71 months randomly selected for the intervention from a rural area prone to vitamin A deficiency and able to complete blood sample. Control Condition: 71 matched children.	Village wide intervention involving seminars focusing on the importance of vitamin A and complementary feeding, distribution of seedlings for vitamin A rich produce (guava, pawpaw, vegetables), establishment of school gardens, promotion of homestead gardens and agricultural training.	Blood samples, FFQ, stool samples	Five years after implementation, 65% of children in the intervention area consumed vitamin A-rich foods more than 7 times a week, compared to 37% in the control area (P = 0.001). Greater frequency of green vegetable consumption was associated with increased serum retinol (Spearmans rho = 0.21, p = 0.01). Children in the control area had higher mean serum retinol (19.4 ± 9mg dL) than those in the intervention area (14 ± 8mg dL) (P = 0.0001). Differences in serum retinol between the experimental and control areas was no longer statistically significant when adjustment was made for helminth infection.

Two interventions that were implemented and assessed by HarvestPlus found increased programme intensity and duration did not influence results [19–20]. The relationship between OFSP intake and serum retinol was less clear. In the pilot study for OFSP a significant 22% reduction in children diagnosed with Vitamin A Deficiency (VAD) was found after 2 years, as well as significantly higher intakes of calories and other essential vitamins [18]. However, analysis involved just 38% of children within the intervention group. Also, although the prevalence of VAD dropped from 60% within intervention children at baseline, 38% of measured recipients remained classified as VAD after 2 years [18]. Seasonal availability could be influencing this result as the percentage of children consuming OFSP 3+ days/week dropped from 54% to 8% within the intervention group outside of harvest season [18,20]. A follow-up HarvestPlus study found a significant 9% reduction in the percentage of children with low serum retinol (<1.05) after 2 years [20]. Though no difference in serum retinol was found for women, or children with diagnosed VAD. Assessment was also limited to those with low serum retinol at baseline in order to maximize sensitivity [20]. All studies highlighted the significant impact vitamin A supplementation had on serum retinol levels, though low levels of national coverage hindered impact.

Analysis of a broader vitamin A community bio-fortification project found that significant changes in dietary behaviour remained, notably increased vegetable consumption, five years after implementation [21]. However, the impact of these behaviour changes on nutrition status was hindered by the presence of helminth infection. As a result, no significant differences between intervention and control serum retinol were found.

#### Social protection programmes

Eight studies assessed social protection programmes implemented to prevent food insecurity in areas affected by economic crisis (Table 3) [15,22–26,42–43]. As they focused on preventing undernutrition, the majority of papers focused on measurement of stunting, body weight and calorie intake to assess calorie deficits, as well dietary diversity to assess dietary quality.

**Table 3:** Social protection programmes impact on diet.

Study Population (n)	Intervention	Outcome Measures	Outcome
**Langendorf, 2014, Niger [22]**			
4176 children aged 6–23 months living in Madarounfa health district which is a rural area with a high poverty rate. Control Condition: No formal control group as all target children had moderate acute malnutrition (MAM).	Evaluated the effectiveness of different combinations of cash transfers in combination with supplementary foods over 5 months to prevent moderate acute malnutrition (MAM) and severe acute malnutrition (SAM).	Anthropometry, edema, mortality	The incidence of MAM was two times lower in children receiving a food supplement in combination with cash, compared to the cash-only strategy (cash versus high-quantity lipid based nutrient supplements (LNS)/cash adjusted hazard ratio [HR] = 2.30, 95% CI 1.60–3.29; cash versus Super Cereal(SC) +/cash HR = 2.42, 95% CI 1.39–4.21; cash versus Medium-quantity LNS/cash HR = 2.07, 95% CI 1.52–2.83) or the supplementary food only groups (High-Quantity LNS versus High-Quantity-LNS/cash HR = 1.84, 95% CI 1.35–2.51; SC + versus SC+/cash HR = 2.53, 95% CI 1.47–4.35). The incidence of SAM was also three times lower in the SC+/cash group compared with the SC+ only group (SC+ only versus SC+/cash HR = 3.13, 95% CI 1.65–5.94). For groups receiving food supplements + cash the incidence of MAM correlated to the calorie content (SC+/cash: 820cal = 3.33 per 100 child-months, High-Quantity-LNS/cash: 500cal = 3.73 per 100 child-months, Medium-Quantity-LNS/cash: 247 cal = 4.28 per 100 child-months.
**Moench-Pfanner R, 2005, Indonesia [42]**			
15611 mothers and 7429 children from 1500 a mixture of urban and rural poor beneficiary households. Control Condition: 12199 mothers and 5198 children from a mixture of 1500 urban and rural poor non beneficiary households.	Aimed to access the impact of a food-for-work (FFW) programme on anaemia prevalence in mothers and children. Following the Asian economic crisis and Indonesian drought, 5 different NGOs implemented food-for-work programmes which offered different types of work (rehab of infrastructure, agriculture, training) in exchange for food (primarily rice).	Blood sample using HemoCue, Vitamin intake collected using semi-quantitative 24-VASQ	In rural households 86–96% of households reported ‘only consuming’ the food provided, this was much lower in urban poor households (44–50%) as they reported sharing more of the food. Within urban poor households only one area showed a significant increase in expenditure on animal foods (p<0.001). Only among urban poor mothers in Surabaya were the odds of anaemia at end line lower when participating in the FFW programme (OR 0.60, 95%CI [0.40–0.89], p = 0.011), in the same area the control group showed a significant reduction in expenditure on animal foods since baseline (8.5% to 7.7%, p<0.001). Surabaya was also the longest running programme (18months) and the only one to include pinto beans and nutrition education. For children and mothers diagnosed as anaemic at baseline the odds ratio of being anaemic at end line was significantly greater in all areas. There was no reduction in the odds ratio of anaemia for women in other areas, or children in all areas. No subject groups showed a difference in vitamin A intake.
**Marquis GS, 2015, Ghana [23]**			
179 children aged 2–5 years (from two study areas within 3 agro-ecologic zones (Coastal Savannah, Savannah-Forest Transitional, and Guinea Savannah) with active livestock activities. Control Condition: 142 matched control children from the same area. Plus 287 comparison children from other areas.	16 month trial to investigate the impact of participation in an entrepreneurial and nutrition education intervention with microcredit which aimed to promote the consumption of animal source foods on nutritional status of children.	Household food security (adapted from USDA Household Food Security Core Module with emphasis on animal food sources), anthropometry.	The intervention group only showed a significant impact on weight-for-age (-0.88 ± 0.09, p<0.001) and body mass index-for-age (-0.03 ± 0.08, p<0.001) at 8 months. Though all groups showed a reduction in BAZ from baseline (due to drought season), the severity of this decline was lower in the intervention group. No significant difference in dietary diversity between groups (mean 4.8 ± SD 2.2), intervention children showed a 20% higher frequency of animal source food intake (mainly fish) (p<0.001).
**Weiser SD, 2015, Kenya [24]**			
Convenient sample of 140 HIV positive subjects ages of 18–49 years with access to farmland and surface water, evidence of moderate to severe food insecurity and willingness to save the down payment for the loan. Control Condition: 68 control subjects who were judged eligible for the study at 1 year follow up.	A one-year pilot randomised controlled microfinance intervention to improve agricultural output, income and health outcomes for people with HIV. The Shamba Maisha intervention had 3 elements. Microfinance loans- participants saved 6 USD then were loaned 150 USD to purchase farming tools and a water pump (had to repay in 1 year) to improve irrigation of crops, received 8 training sessions on agricultural and financial management.	Food Security (measured by Household Food Insecurity Scale), Diet quality (World Food Programme consumption score, household wealth, anthropometry, HIV RNA.	Food insecurity decreased progressively in intervention and control groups (with a steeper decline seen in intervention), at 12 months’ the difference between intervention and control was -3.685 ± 1.2, p<0.001. Frequency of food consumption was also greater in the intervention group at 12 months (Difference-9.437 times/week, p = 0.013). There was no significant difference in BMI (0.355, p = 0.114) and food expenditure (+220.3Ksh, p = 0.398), however both indicators were significantly higher in the control group at baseline (BMI<18.5: 13(18) vs. 5 (7), p = 0.054). Over 12 months the percentage of virologically suppressed participants in the intervention group rose from 51% to 79%- a 33% improvement in comparison to controls (OR 7.6[95%CI 2.2–26.8],p = 0.002).
**Morris SS, 1999, Honduras [15]**			
Small-holder farm households living on under $US 2000/year from two communities (196 households from the community commencing in 1996 and 193 from the community starting in 1997). Control Condition: 189 matched small-holder farm households from a third community.	To evaluate the impact of a rural development project on household food security and nutrition. Involved the provision of technical training and support in agricultural production to encourage crop diversification, marketing training, the provision of credit for agricultural investments and infrastructure to support.	Calorie intake, maize production, dietary diversity (FAO guidelines).	Two years after the intervention participating farmers had greater maize stores than controls (329%-371% increase vs. control groups 256% increase, p = 0.01. There was no increase in dietary calorie consumption (difference of -1% (-36 calories)- +1%(44 calories) vs. controls -8% (271 calories), p = 0.13). No significant increase in dietary diversity was noted +11%-+9% vs. control group -3%, p = 0.10). The impact on under 5 nutrition was complex.
**Nair N, 2013, India [25]**			
528 rural households which included 1056 individuals. All included households had children aged 1–12 months in the state of Rajasthan.	Aimed to investigate the impact of Mahatma Gandi National Rural Employment Guarantee Act (MGNREGA) on infant malnutrition. Households benefiting from MGNREGA benefited from 100 days of paid employment for manual labor.	Focus Group Discussions, anthropometry, Food and Nutrition Technical Assistance-2 indicator.	No significant difference was found in dietary diversity scores between intervention and control households (5.8[0.10] vs5.7[0.10], p 0.292). Intervention households were less likely to have wasted (OR 0.57,95%CI 0.37–089, p = 0.014) and underweight (OR 0.48,95%CI 0.30–0.76, p = 0.002) infants. Pathway analysis suggested household food security did not impact on infant nutrition, but may impact on birth weight.
**Mascie-Taylor CGN, 2009, Bangladesh [26]**			
Adult females from 895 intervention and 921 control households containing one child under 5. Taken from a total of 100000 households enrolled in the Chars Livelihood Programme (CLP) in northwestern Bangladesh in an area prone to annual floods, unemployment and seasonal food insecurity. Over 50% of women had a BMI <18.5.	Aimed to assess the impact of a cash-for-work programme within CLP households on food insecurity. The intervention involved the provision of wages for labor intensive earthmoving tasks. These tasks resulted in raised land for all CLP households to establish homestead gardens.	7 day FFQ, structured household surveys, anthropometry.	Compared to control households, intervention households reported a significant increase in food expenditure on cereals, pulses, green leafy vegetables (GLV), eggs, fish, meat and oil (p<0.001 and a significant increase in consumption of all foods except cereals. The control group reported a reduction in consumption in all food groups. Notably intervention households reported a 36.5% increase in GLV intake, while control households saw a 35.4% reduction. Intervention households showed a higher proportion consuming GLV (24.9% vs. 5.6%, p<0.001) on 7+ days a week and fruit on 3+ days a week (3.9% vs. 0.8%,p<0.001). At end line significantly fewer women had a BMI <18.5 (48.4% vs. 56.6%, p<0.001). In children, more intervention children improved from being underweight to normal weight (7.3% vs. 3.3%.
**Asfaw A, 2007, Egypt [43]**			
Population wide model based on 902 mothers in Egypt, which faces some of the highest rates of obesity. Control Condition: No control population.	Egyptian food subsidy programme aimed at reduced undernutrition, infant mortality and reduce the impact of economic shocks. Subsidies are available for bread (57%), wheat flour, sugar and cooking oil for poor and rich households.	Results from the 1997 Egyptian Integrated Household Survey including expenditure and anthropometry.	The average BMI was 27.6 (SD 6.30). There was no significant difference in overweight and obesity between extremely poor and non-extremely poor households. Bread and sugar showed significant impacts on BMI. Bread elasticity on mothers BMI is 0.119 (SD 0.047, p<0.05)) (1% price increase would lead to 0.119 BMI reduction. For sugar too (-0.112 (0.054, p<0.05)) a 1% increase in price of sugar for every 100 calories could decrease mothers BMI by 0.11%, and rice -0.203 (0.074, p<0.001). The reverse for fruits 0.09(0.037, p<0.05), eggs and milk 0.137(0.045, p<0.001). Despite bread, sugar and oil contributing to just 4% of expenditure, they constitute 31% of total calorie availability.

An examination of varying methods of delivery for cash transfers found those delivered in combination with supplementary foods were twice as effective in preventing undernutrition within children, compared to cash in isolation [22]. The decline in moderate undernutrition increased as the calorie content of supplementary food increased. A large study examining the impact of food-for-work programmes on anaemia found no impact on anaemia for children or women in rural settings [42]. Only a small improvement in anemia was seen within one subset of urban women. In comparison to other settings, the women in this urban area received pinto beans (in addition the rice and oil received by other groups), had the longest programme participation and received direct nutrition education [42].

A small but significant protective effect on childhood undernutrition was identified for a microcredit programme in Ghana, which incorporated nutrition education, though it was not sufficient to overcome an overall reduction in child weight and BMI due to a prevailing drought [23].

The intensive Shamba Maisha project in Kenya supported improved agriculture through conditional loans requiring health clinic visits for HIV sufferers [24]. The project found a significant reduction in underweight and food insecurity. Another agricultural loan intervention in Honduras found no change in calorie intake despite increased crop stores [15]. Two programmes investigated the impact of creating paid employment opportunities on nutrition. Both identified a positive impact on children’s nutrition status- reducing levels of undernutrition [25] as well as increasing household food expenditure and consumption–particularly of green leafy vegetables [26]. Social protection programmes comprised of direct nutrition education, provision of nutrient rich food, and longer duration were associated with greater impact on diet.

In Egypt, an economic analysis of the impact of government food subsidies on national obesity levels found a significant association between reducing bread, wheat, flour, sugar and cooking oil prices and increased female obesity levels. Conversely, subsidies on healthy foods were modeled to reduce BMI [43].

#### Agricultural diversification

Eight studies examined the impact of agricultural diversification interventions on dietary outcomes (Table 4) [27–34]. Two involved implementation of a Helen Keller International homestead garden programme. One before/after evaluation utilising cross-sectional surveys found a significant increase in nutritious food consumption (notably vegetables) after programme implementation, that did not equate to improved nutrition status [27]. The second, larger RCT identified an improvement in hemoglobin levels in young children [28]. Results from this study may have been influenced by hygiene improvements which were also promoted during the trial and could have reduced infection, another cause of low haemoglobin.

**Table 4:** Agricultural diversification interventions impact on diet.

Study Population (n)	Intervention	Outcome Measurement	Outcome
**Olney DK,2009, Cambodia [27]**			
Cross-sectional sample of 300 households at baseline and end line. Households had a female responsible for agriculture, and a child aged under 5 years. Control Condition: Cross-section of 200 matched households at baseline and end line.	To project aimed to evaluate the impact of a two year Helen Keller International homestead gardening project on maternal and child nutrition status in low socio economic households. The intervention promoted homestead food production via the provision of training, tools and management assistance.	Anthropometry, blood sample, FFQ	At end line, a greater proportion of households in the intervention group consumed dark-green leafy vegetables (95.0% vs. 87.5%, p<0.05), yellow or orange fruit (71% vs7% vs. 63.0%, p<0.05) however the volume consumed (in kgs) was not different. There were no differences in hemoglobin, weight or BMI.
**Olney DK, 2015, Burkina Faso [28]**			
884 Households within 55 villages in Gourma. Average of 7–8 members per household, 88.9% anaemia in children, 31% stunted,38% underweight, 38% wasted. Control Condition: 597 households in the area not partaking in the intervention.	Involved two year Helen Keller International (HKI) homestead food production and nutrition and health behaviour change communication (BCC) program. Agriculture production activities included input distribution (e.g., seeds, saplings, chicks, and small gardening tools). Two intervention arms, 1) intervention components delivered by older women leaders (OWL) 2) delivered by health committee.	household surveys, structured dietary interviews and clinical assessments	In children aged 3–5.9 the only significant improvements were seen in the HC group with an increase in Hemoglobin levels (0.76 ± 0.33 g/dL, p = 0.02) and reduction in anaemic children (-14.6pp, p<0.02) compared to controls. In children aged 3–12.9 months a significant reduction in diarrhea prevalence was found in the OWL (-9.8pp, p = 0.05) and HC groups (-15.9pp, p = 0.00).
**Hanson M, 2011, Federated States of Micronesia [29]**			
A sample of 40 residents of Kapinga Village which has a high level of food insecurity and poverty. Control Condition: baseline measures taken from 68 participating households.	Aimed to investigate the impact of an NGOs ‘Go Local’ food promotion programme on dietary intake. The intervention involved 3 components. 1) Agricultural training and the provision of seeds to encourage container gardens. 2) Charcoal oven workshop and promotion 3) nutrition education sessions focusing on eating healthy, local foods.	Household surveys, FFQ	One year after the intervention the mean days’ local fruits were eaten per week increased (2.8 ± 2.2, 4.6 ± 2.0, p<0.001), along with local vegetables (1.2 ± 1.6, 2.9 ± 2.5, p<0.001) and local fish/seafood (2.5 ± 2.6, 4.4 ± 2.2, p<0.001). Imported fruits, vegetables and drinks with sugar also increased.
**Kariuki LW, 2011, Kenya [30]**			
5 community women’s groups (actual participation numbers not specified) within the Kuiti district of Kenya. Control Condition: no control group, comparison made to baseline measures of participants.	The intervention involved the distribution of seeds, nutrition and cooking classes, technical agricultural support and the establishment of market linkages to promote 8 local, micronutrient rich vegetables which were not commonly consumed in the area. Different women’s groups received differing levels of programme intensity/coverage.	Participant group survey	By the end of the one-year period, two species had been adopted for both consumption and marketing but to different extents. The groups that received all interventions, showed the greatest increase in consumption and selling of the crops. During the first half of the year, African nightshade got into the market for the first time. Within the same period there was a noticeable increment in sales of leaf amaranth. Acceptance of spiderplant, both for home consumption and marketing, lagged behind and only started picking up at the end of the one-year intervention period. An important lesson learnt is that the five intervention strategies are complementary and useful in the promotion of underutilised species.
**Bamji MS, 2011, India [31]**			
Convenient sample of 178 Farmers (from a total baseline sample of 222) from 15 villages of the Medak district of the South Indian state of Andhra Pradesh. Control Condition: 50 Farmers from the same region.	Investigated the impact of partial crop diversification on household’s access to vegetables over a 3-year period. The intervention involved the promotion of diversifying from water based rice and sugar cane production to green methods of farming which produce vegetables via the provision of seeds, organic fertilizer, agricultural training and nutrition education.	Knowledge, Attitude and Practice surveys, semi-quantitative diet survey	Vegetable intake was higher for intervention households compared to control (52.3 ± 21.7 vs. 37.1 ± 10.34 g/capita/day, p, 0.05), Green leafy vegetable intake was higher in intervention compared to baseline (51.6 ±24.3 vs. 57.1 ± 24.4 g/capita/day, p = 0.05). Improvements were also seen in egg and meat consumption. Despite improvements, intake of vegetables and animal source foods remained low.
**Singh H, 2014, India [32]**			
Fifty farm families selected from Suhagheri village. Control Condition: No control group- only baseline comparison conducted.	Aimed to assess the impact of crop diversification on farmer’s income and intake of nutritious foods in place of traditional cereals. The intervention involved the introduction of pulse, fruit and vegetable crops in the kitchen gardens of farms previously solely producing rice and wheat. Crop diversification choices were made based on the results of water and soil tests of the area.	Crop production, crop consumption (method not reported), self-reported kitchen gardening practices	Intake of vegetables increased by 165g (55g green, 65g leafy vegetables), fruits by 10g and pulses by 35g. The practice of kitchen gardening within the village increased from 36.4% to 84.4% after the trainings, with 1.5 hours more spent on gardening in the new crop areas. As a whole, involved farmers reported producing approximately Rs 14296 ($US 226) worth of pulses, fruits and vegetables by growing their own vegetables which translates to cost saving as they did not need to purchase those products at the markets. No statistical analysis conducted on these results.
**Sharma KR, 1999, Nepal [33]**			
112 boys and 144 girls aged 6–36 months from farming households selected to participate in the VFC programme. 44 households were selected from 3 diverse regions (Satabariya, Jinabang, Thabang) in Nepal, all of which traditionally relied on subsistence farming. Control Condition: 121 boys and 134 girls aged 6–36 months from 44 matched households from 3 the different regions.	Evaluated the impact of farm commercialization programmed known as VFC on nutritional status of children 5 years after implementation. The intervention involved the promotion of vegetables, fruits, and cash crops (VFC) via the provision of seeds and tools, trainings and technical assistance aimed at increasing rural incomes through promoting market orientated agricultural production. Implemented in Nepal since 1985, data collected between 1991–1992.	Household demographics, socio-economic measures, anthropometry	Results from a simple ANOVA test suggested that participation in VFC was associated with improvements in the weight-for-age (F-value 17.83 (-1.39 vs. -1.91), p<0.01) and weight-for-height (F-value 20.96 (-.033 vs. -1.61), p<0.01) for boys, though no significant differences between control and participant households was found for girls. When results were controlled for other determinants of child nutrition the VFC share of household income was not associated with children’s nutrition status. Significant determinants on the nutritional status of children included household size, mother’s education level, mothers’ time in agriculture, mothers BMI and child age. Though VFC households showed a higher income than controls, this did not correlate to child nutrition status.
**Jones KM, 2005, Nepal [34]**			
430 farming households in the agricultural Lumbini-Gandaki region. Control Condition: 161 farming households who took part in vitamin A awareness training but not kitchen garden project. 389 matched control households not partaking in the project.	To evaluate the project’s impact on increasing production of high-value-crops and improving the nutrition status of participants. The Market Access for Rural Development (MARD) project involved the establishment of kitchen gardens (including seed provision, training and technical assistance) and nutrition education. The intervention focused on the production of high-economic-value crops.	Nutrition knowledge questionnaire, self-report practices, diet recall	After 36 months a significantly higher proportion of intervention mothers reported changing their diet during pregnancy (91.8 vs. 82.8, p<0.005), household production for consumption of all macronutrient rich fruits and vegetables were also significantly higher (p<0.001)—in particular self-reported consumption was significantly higher than controls for green leafy vegetables (broad leaf 40%, fenugreek 30%, amaranth 40%, spinach 50%), carrot (58%) and ripe mango (30%).

Two small pilot studies found the promotion and cultivation of local fruits and vegetables had the potential to increase consumption and sales [29–30]. This was consistent with two crop diversification projects which found an increase in vegetable intake, though not to a high enough level to reach requirements [31–32]. Two studies from Nepal involving interventions to increase market access and sale of cash crops showed improvements in crop production, including fruits and vegetables, as well as increases in vegetable intake [33–34]. These did not have a significant impact on childhood underweight when controlled for other factors [34].

#### Livestock and fisheries management interventions

Three studies examined the impact of improved livestock and fisheries management on income and nutrition status (Table 5) [35–37]. Two focused primarily on dairy production (including the introduction of milk producing cows, and dairy management training) [35–36]. One showed an increase in dietary fat and iron intake [35] while the other reported an increased intake of milk and green leafy vegetables [36]. They also reported on potential indicators of over consumption with one reporting an increased calorie intake to over the recommended 2000 calories a day [35], and the other reporting an increase in obesity [36]. The remaining livestock intervention focused on the improved management of aquaculture ventures in Bangladesh which resulted in significant increases in fish consumption [37]. All livestock interventions resulted in an increased consumption of animal source foods. No studies adjusted for confounders.

**Table 5:** Livestock and fisheries management interventions impact on diet.

Study Population (n)	Intervention	Outcome Measures	Outcome
**Ahmed MM, 2000, Ethiopia [35]**			
84 farm households from Holetta area, selected using convenience sampling. Required sufficient finances to fund maintenance costs. Control Condition: Sixty control households using traditional practices for milk production which matched the wealth groups of intervention households.	Aimed to quantify the impact of new dairy technologies on household income, expenditure on food and nutrition. The intervention involved introducing crossbred cows plus complementary feed and management technologies to increase dairy production in smallholder farms. Half the cows were used for milk production and half for traction and milk production.	24 hour dietary recall, Household surveys	Total monthly income was significantly higher in intervention households (225 vs. 131 USD, p<0.05), so too was per capita food expenditure (14.9 vs. 12.4 USD, p<0.05). Dietary intake of calories (2332 vs. 1959 kcal), Fat (19.6 vs. 15.8 g), protein (70.3 vs. 62.1g), retinol (38.8 vs. 27.1 μg) and iron (74.2 vs. 65.6μg) were all significantly higher (p<0.05) in the intervention group. The number of crossbred cows owned was significantly associated with income (parameter estimate 0.0643) and calorie intake (parameter estimate 0.0236), p<0.001.
**Walingo MK, 2012, Kenya [36]**			
The female heads of 150 intervention households in the Vihiga District where 60% of the population lives in poverty. Recruited from women’s groups in the region. Control Condition: Women from 150 non-beneficiary households who did keep livestock.	Aimed to quantify the impact of the Livestock Development Programme (LDP) on socioeconomic and dietary outcomes. During the intervention participants were trained in basic dairy management skills and were provided with chuff-cutters, rain water catchments roof tanks all designed to reduce drudgery.	24h Diet record, structured interview and anthropometry	Intervention households scored significantly higher on food and nutrient intake meeting recommended dietary intakes set by the FAO (33.54 ± 10.20 vs. 28.45 ±9.43, p<0.001). Both milk and milk products and leafy green vegetables were found to be higher in intervention households (Mahalanobis D^2^ 3.17, discriminant factor 2.35, F-Ratio 14.46). Prevalence of obesity was higher in intervention women (6% vs. 4%)—BMI was associated with sale of harvested crops and ability to purchase staples. Nutritional status showed no significant improvement and increased income was not spent on food (instead it was invested back into dairy farming).
**Ahmad KM, 2010, Bangladesh [37]**			
225 farmers from four Development of Sustainable Aquaculture Programme (DSAP) areas, selected to represent different wealth ranks (judged by land holding). Average age 40, with 7 years’ formal education. Control Condition: Baseline data from farmers before commencing DSAP, plus 123 farmers not involved in DSAP.	The intervention involved 3 years of continuous aquaculture training support to farmers utilising a Participatory Adaptive Learning (PAL) approach which included training for the whole family on improved pond management. The intervention aimed to increase income and food security.	Household demographics, income, fish production, sales and consumption	In comparison to controls, DSAP farmers saw 19.1% increased growth in fish production (mean difference 9.9%, p = 0.01), and 737kg/ha more fish sold (mean difference 9.5%, p = 0.05). The average fish consumption in DSAP farmers saw a significant increase of 293g/capita/month between 2003–2006 (6.6% growth, p = 0.01). This was significantly higher than control farmers (mean difference 4.6, p = 0.01). DSAP farmers also experienced increased net income from fish culture (139 USD vs. 62 USD, p = 0.01).

#### Multi-component programmes

Four programmes involved multi-faceted interventions addressing development indicators which measured dietary outcomes (Table 6) [38–41]. One was a population level analysis which aimed to examine the impact of Vietnam’s VAC programme which encouraged nationwide adoption of traditional agriculture techniques to improve the diversity of farmed crops and livestock [41]. The impact of this national initiative was evaluated by extrapolating results of the national nutrition survey which suggested an increase in dietary calories, fat, protein and a reduction in VAD [41].

**Table 6:** Impact of multi-component programmes on diet.

Study Population (n)	Intervention	Outcome Measures	Outcome
**Hop LT, 2003, Vietnam [41]**			
Nationwide evaluation. Control Condition: Baseline data from 1987.	To investigate the impact of the V (Vuon for garden), A (Ao for pond) and C (Chuong for cattle shed) programme implemented by the National Institute of Nutrition which aimed to diversify agricultural products. Encouraged traditional Vietnamese farming systems through the distribution of land for farmers to grow diverse products (rather than only rice), many different models of VAC are implemented by making loans to poor families.	FAO Food Balance sheets, National Nutrition Surveys, UNICEF child health surveys	Between 1965 and 2000 total calorie intake increased from 1872 to 1931, the % of total energy from fat increased from 7 to12 and protein from 10 to 13.2. Between 1985 and 2001 the prevalence of underweight reduced from 59.7 to 34.8 and stunting from 51.5 to 31.9. From 1995 vitamin A deficiency reduced from 14.5% to 10.2%. The prevalence of anaemia also declined in women and children. Food production has seen increases in animal sources, legumes, rice and oil.
**Ekesa BN, 2013, Burundi, Democratic republic of Congo, Rwanda [38]**			
Farm household within 5 villages from 7 mandate areas- selected for analysis due to intensity of CIAlCA programmes. Control Condition: Control villages selected due to lack of CIALCA product promotion.	To evaluate the impact of improved farming technologies on self-perceived food security and dietary diversity. Intervention involved distribution of technologies to improved legume and banana/plantain varieties, plant disease management, profit enhancing and quality management technologies. Guidelines on and marketing tools were also developed. Development partners in ‘satellite sites’ also disseminated technologies.	Perception of food sufficiency and quality, 24 hour dietary recall	No significant difference in calorie intake was identified between intervention and control groups, 53% of respondents in control sites indicated a decrease in intake of protein rich foods, this was significantly higher than the proportion in the action (46%) and satellite (41%) sites, p <0.05, suggesting they were slightly protected from drought conditions.
**Abebaw D, 2010, Ethiopia [39]**			
200 Kebels of which 100 were in Ibnat and the other 10 in Belessa. Control Condition: Predicted outcome if the programme had not been implemented.	Aimed to predict the impact the Ibnat–Belessa integrated food security programme (IFSP) has had on calorie intake. The IFSP intervention integrates environmental rehabilitation, water supply, irrigation, livestock, crop production, fruit and vegetable production, feeder road construction and maintenance	Household questionnaire, structured interview	Modelled Food calorie intake predicts a 30% increase in calorie consumption per adult/day (1773 to 2425, adjusted impact estimate, 695 ± 4.77, p = 0.01). Land rich households with female heads and small family size benefited more from the intervention.
**Remans R, 2011, Ethiopia, Ghana, Kenya, Malawi, Mali, Nigeria, Senegal, Tanzania, Uganda [40]**			
2700 households (from 8,652 surveyed) from 9 project sites drawn from hunger hot spots: Ethiopia, Ghana, Kenya, Malawi, Mali, Nigeria, Senegal, Tanzania, Uganda. High level of poverty and farming was the main source of livelihood. Control condition: baseline measures	The Millennium Village Project (MVP) is a multicounty, multisector rural-development initiative which involved a package of evidence-based interventions in agriculture (subsidised fertilizers, improved seeds, livestock rearing, fish farming, food processing, trainings) health (supplementation-iron, folate, vitamin A, growth monitoring, deworming, treat severe malnutrition, bed nets, CHW), education (breastfeeding promotion, complementary feeding, nutrition education), and infrastructure sustained (construction of water sources, road improvements, cook stoves, mobile phones for emergencies) over a 10-y period.	FFQ, Anthropometry, FAO Diet Diversity and Food Security Score	After 3 years’ significant improvements were seen in Number of meals (adj OR 1.30 (1.11, 1.52), dietary diversity (adj OR, 1.25 (1.02, 1.52), Stunting (adj OR 0.57 (0.38, 0.83) and underweight (adj OR, 1.18 (0.71, 1.96). Significant improvement in height for age z-scores in Ethiopia (-2.34 ± 1.91 vs. -0.96 ± 2.45, p<0.001), Kenya (-2.46± 1.94 vs. -1.18± 2.51, p<0.002), Tanzania (-1.52± 1.89 vs. -.52± 1.67, p<0.001) and Mali (-2.47 ± 2.25 vs. -1.19± 1.86, p<0.006).

Two studies evaluated the impact of improved agricultural technologies on crop outputs and food security [38–39]. In one, no difference in self-reported calorie intake was found for programme recipients, though protein intake showed a smaller reduction than was reported by control participants as a result of prevailing droughts [38]. The other was a modeling study which predicted that a food security programme focused on improving water irrigation for agriculture would result in a 30% increase in available calories (695cal/day) [39].

The final study evaluated the impact of the Millennium Development Project in nine settings within low income AFRO countries [40]. While significant improvements were seen in the number of meals, dietary diversity and stunting–these may have been influenced by the use of community health workers during implementation as opposed to solely agricultural and poverty reduction components [40]. The use of total dietary diversity scores mean we are unable to quantify increases in specific food groups.

#### Obesity

Within the 25 studies that met eligibility criteria by including outcome measures related to unhealthy diets, eleven studies also included a measures of calorie intake and body mass index in adults [15,18,23–27,35–36,39,43]. These measures can be used to assess overconsumption and obesity, both risk factors for NCDs. In the six studies which measured calorie intake [15,18,35,38–39,41], four identified an increase [18,35,39,41]. Two of these studies found an increase to over 2000 calories [35,39], both were based in Ethiopia and involved the promotion of livestock projects in participants involved in agricultural work. Six studies evaluated BMI with two agricultural diversification projects finding no impact [23,27], while four showed an increase [. Two studies reported only on a reduction in underweight participants [24,26], two showed an increase in obesity when evaluating livestock management programmes [36] and government food subsidies [43], both of which aimed to improve food security. No agricultural diversification projects showed any significant increase in calories or body weight.

### Quality assessment

Application of quality assessment tools identified several common weaknesses in the included studies. RCTs (S4 Table) rated as ‘unclear’ in relation to their level of reporting bias as they tended to suit the description of observational trials more than RCTs [19,20,24,28]. In particular, there was no reporting on the process of randomization or allocation concealment and study design meant participant/ researcher blinding was not possible. Both bio-fortification trials also lost over 10% of participants within intervention arms influencing outcome data [19,20]. They were also all linked to, or were conducted by the organization providing OFSP which could introduce reporting bias [18,19,20]. Weiser et al (2015) was conducted independently from the World Food Program funders and showed lower risk of bias, aside from the inability to blind participants/researchers and the possibility of cluster level variables which were not adjusted for [24].

Cohort and cross-sectional studies also showed common limitations. This was particularly apparent in their use of convenience sampling of highly motivated, high needs, easily accessible programme recipients- limiting the generalizability of results (S4 Table). Many also experienced a high attrition rate, or failed to record the attrition rate which could mean the true impact is lower than reported. As many measured dietary intake of food groups, there is also likely to be great variation between studies in this measure, with measures including self-report, self-completed FFQs, guided FFQs, structured interviews, diet recall and dietary diversity measurement using pre-validated forms. While cluster level differences, household characteristics and infection all proved to be important confounders, many studies did not adjust for these factors. Ten papers made no adjustments for confounders and only two scored highly for controlling for confounders (S4 Table).

Overall, studies led by university groups as opposed to development partners tended to report lower magnitudes of impact, adjust for potential confounders and discuss limitations.

## Discussion

This review highlights the paucity of NCD related outcome measures within the design and evaluation of current development interventions within LLMICs. Very few papers included outcome measures or objectives which complemented global NCD prevention strategies which focus on reducing unhealthy behaviours, and implementing population based strategies [8,9].

For example, the paper relating to NCD morbidity was in relation to arsenic as opposed to the four modifiable behavioural risk factors [16]. The study addressing physical activity also aimed to reduce exertion as opposed to reducing sedentary behavior [17], while dietary related papers focused on the reduction of undernutrition as opposed to overnutrition [18–43].

Only 12 studies reported on outcomes consistent with the global NCD indicators stated within the NCD GAP [8], this included two on obesity and ten on fruit and vegetable intake. No studies reported on the global NCD indicators relating to the intake of saturated fat, salt, physical inactivity, alcohol or tobacco use. Seventeen studies reported on other dietary measures including undernutrition (via dietary diversity, micronutrient deficiencies and underweight), fortified crop intake, mitigating water contamination and one on reduced physical exertion from manual labor.

Overall, agricultural programmes which focused on bio-fortification and diversification of crops showed a positive impact on the consumption of nutritious foods, particularly vegetables. That said, this impact was often not sufficient to meet the WHO 400g/day recommendation for fruits and vegetables [8]. Additionally, these programmes showed inconsistent impacts on nutrition status measured primarily by vitamin A deficiency, anaemia, stunting and body weight in women and children. These mixed findings of impact on the nutrition status of women and children are supported by a previous review into the impact of nutrition-sensitive interventions [44]. With 81% of included studies involving farming households, there is also the risk that potential NCD risk factors which fall outside the primary four, have been missed. For example, agricultural projects which focus on increasing agricultural output and income via tobacco crops could unintentionally increase tobacco usage [45]. Exposure to residues from agricultural pesticides through agricultural work has also been found to increase the risk of CVD [46]. Agricultural interventions could therefore place participants at a higher risk of NCDs despite increasing their vegetable intake.

The high number of studies evaluating the impact of agricultural work interventions reflects the focus of many development interventions, especially within Africa, due to agricultures immediate impact on food security and long term benefits for economies. The physically demanding nature of agricultural work indicates many participants involved in the studies would have ample daily physical activity and high calorie requirements. It is therefore unlikely they would constitute the target population of interventions aimed at increasing physical activity. This possibility is reflected in this review’s findings of just one physical activity paper that aimed to reduce physical exertion in physically fit hill farming women [17]. It is also supported by another social protection programme which noted that the increased physical exertion brought on by employment raised concerns that the intervention may exacerbate undernutrition [25].

While not searched for specifically, an important finding within eligible studies was the association between increased access to food through (improved livestock management, provision of supplementary foods or improved affordability of nutrient poor foods, as a result of poverty reduction interventions) and increased prevalence of obesity. These findings complement findings from social protection programmes in Mexico and Brazil which have found an increase in obesity for recipients of food assistance programmes [47–48]. Within our review, cash transfer was also shown to increase body weight in undernourished children proportionate to the calorie content of supplementary foods [22]. The pattern of increasing purchasing power, supplementary foods and obesity raises an important consideration for future projects, which must consider the quality of foods as well as food access, in order to prevent contributing to the double burden of nutrition. This is an essential point of focus for future development programmes as it may supersede other noted health benefits [49].

Traditional measurements of diet within food security programmes could also contribute to this problem as they focus on calorie content over nutritional quality of available food. For example, dietary diversity scores are intended to measure diet quality by reflecting the number of different food groups consumed [50]. While reporting the total score can give an indication of improvements in diversity and provide a comparative measure–it does not reflect the quantities, nor give an indicator of the nutritional quality of consumed foods. In particular, ‘oils and fats’, ‘sweets’ and ‘spices, condiments, beverages’ are each separate food groups [50]. Hypothetically, an increase in one of these food groups would improve the total dietary diversity score. Reporting on the results of each food group would provide a clearer understanding of impact on dietary composition and nutritional quality. The importance of such considerations are highlighted by recent findings that for every 10% rise in GDP there is a 6% reduction in childhood stunting, and 7% increase in overweight prevalence [44]. With growing evidence that children affected by stunting may be at increased risk of NCDs later in life, it is essential to maximise the diet quality within LLMICs to prevent an escalation of the double burden of malnutrition [51]. Improved reporting and measurement of dietary indicators that reflect both under and over nutrition will be essential in achieving SDG 3 relating to NCDs.

Within included studies the focus on reducing undernutrition, physical hardship and exposure to harmful agents largely reflects the major, immediate health risk factors faced by included LLMICS. For example, in the African region, where the majority of included studies were conducted, the leading health risks are childhood under-weight, unsafe sex and unsafe water [52]. This suggests that while NCDs are a growing concern within the African region, differing risk profiles mean interventions outlined in the WHO Best Buy interventions may not be the most effective or urgent measures in reducing the disease burden.

Reflecting countries stage of epidemiological transition, other regions show a much higher prominence of modern risk factors. For example, in the South East Asia, where nine included studies were conducted, high blood pressure, BMI and smoking are now leading health risks [52]. The same is true for LLMICs within EURO, EMRO, WPRO and PAHO where NCD related health risks are superseding MDG related risks [5,52]. With only 14 studies from these regions identified, findings of this review highlight the lack of research on the synergistic impact of interventions addressing development indicators within LLMICs who face the highest burden of four key behavioural risk factors and consequential NCD outcomes.

### Strengths and limitations

Many papers have reported on the risk of NCDs and behavioural risk factors within LLMICs [53], the impact of NCDs on international development and the need for multi-sectorial action to combat them [8]. This is the first review to assess what impact current development interventions are having on NCDs. It highlights the potential for future development interventions to impact on NCDs with intervention design, NCD sensitive objectives and outcome measurement. Our work complements previous reviews on the importance of nutrition sensitive agriculture and growing evidence of the possible negative impacts food assistance can have on NCD outcomes.

While no language restrictions were placed on included papers, the search was conducted in English, which may have been a limitation as English is an official language in only 35% of LLMICs. Exclusion of grey literature also narrowed the review’s scope, as development agencies may not have published all available results within academic journals. The decision to exclude grey literature was made by the review team in light of the overwhelming number of case-study reports and the tendency for such reports in international development interventions to be subject to outcome reporting bias [54]. This is influenced by a number of factors and generally demonstrates selective outcome reporting which favours the reporting of positive impacts which reflect favourably on the implementing partner and donor [55]. As evident in the studies included within this review, such reports also tend to employ anecdotal evidence without rigorous statistical analysis or peer review, and frequently funded by the programme funder. Many grey literature reports identified show great potential for future academic publications, such as modeling studies investigating the impact of trade agreements and improved human rights on NCD outcomes [56].

Additionally, the use of World Bank Classifications of LLMICs impacted on results, as countries often classified as ‘developing’ such as South Africa, Brazil, Thailand and Mexico, for which a greater number of studies are available, were not included [11]. Our review also failed to find any papers from the European region in which LLMICs face very high levels of NCD risks. Choice in search engines may also have swayed our citation findings towards health centric results.

The nature of development research could also have introduced a systematic bias towards positive results due to the closeness of studies to funders, advocates and political interests [57]. This could introduce publication bias as studies which identified a positive outcome were more likely to warrant time investment in the publication of results. The heterogeneity of papers means we are unable to conduct a funnel plot to assess this bias. There is also the potential for selection bias as interventions address the most at need subjects and many studies experienced a high loss to follow-up, as well as measurement bias in papers such as those evaluating bio-fortification where only children with severe deficiencies were included in analysis. Within our review, the positive impacts on health and development indicators may therefore be over-estimated.

An additional limitation of the review is the focus on specific dietary factors, as opposed to wider nutrition factors. There is growing evidence of nutrition factors such as early stunting and growth on NCD outcomes [58]. Preventing such states of undernutrition has historically been a key focus of development interventions and recent research has shown the great potential of interventions such as cash transfers [49], bio-fortification [59], and improved water and sanitisation [60] programmes to reduce and prevent stunting prevalence.

The descriptive nature of the review, due to the heterogeneity of papers, means we are unable to quantify the impact on NCDs. As many of the included studies, particularly agricultural diversification interventions, involved small sample sizes, convenience sampling of highest need populations and lack of control for confounders they are limited in their generalizability and not representative of national level programme focus. This is compounded by the fact that many countries, including all LLMICs in the European region, are not included in our review.

## Conclusions

While current research on the duel impact of interventions on development, poverty reduction and NCD prevention is scarce, great potential exists for future programmes. It is essential to note that a key finding of this review is the lack of evidence for development interventions on NCD outcomes, as well as the lack of inclusions of NCDs in interventions outcome measures. A historical dominance of development interventions on addressing undernutrition has meant the measurement tools and study designs continue to focus on measures of undernutrition despite the growing double burden of malnutrition and NCDs. Measurement structures and programme focus on undernutrition do however create great potential. The promotion of healthy diets within development interventions is an important area for future focus.

Addressing health and NCDs is essential for interventions to have a sustainable impact on development and achieve the SDG targets. Failure to do so could compound current health problems and hinder development. Setting NCD sensitive objectives and measurements within poverty and development interventions could lead to simple adjustments, with great impact. Results from our review also highlight how NCD risk factors within LLMICS stray from the four commonly targeted behaviour changes. In measuring risk and impact on NCDs within future interventions, it is recommended to tailor programmes to country-specific risk factors. Improved measurement tools and guidance could enhance this process and strengthen multi-sector action and impact measurement to address NCDs within LLMICs. These improvements would also help to standardize reporting and expand the research field. As a growing number of development interventions impact on NCDs to achieve the SDGs, the evaluation of NCD outcomes and publication of results within academic literature is highly encouraged to promote best practice and inform effective resource allocation.

## Supporting information

S1 Table PRISMA checklist. Reviews compliance to PRISMA guidelines.
(DOCX)

S2 Table Search strategy. Full search strategy utilized for literature search.
(DOCX)

S3 Table Inclusion criteria. Measurement and outcome criteria for review inclusion.
(DOCX)

S4 Table Quality assessment. Quality assessment of included papers.
(DOCX)
