# Supplementary material for: The impact of poverty reduction and development interventions on non-communicable diseases and their behavioural risk factors in low and lower-middle income countries: A systematic review
Source: PLoS One. 2018 Feb 23;13(2):e0193378. doi: 10.1371/journal.pone.0193378 (PMC5825092; doi:10.1371/journal.pone.0193378)
Supplement: S2 Table — Full search strategy utilized for literature search. (DOCX) [file pone.0193378.s002.docx]

S2 Table. Search Strategy.

Searches conducted on 15^th^ February 2016. Embase, Global Health and Ovid MEDLINE (R) In-Process & Other Non-Indexed Citations and Ovid MEDLINE(R) databases searched via the OVIDSP interface. Web of Science Core Collection database searched via the Thomson Reuters inferface.

Search Terms and Strategy (example taken from MEDLINE search for NCD Risk Factors)

| # ▲ | Searches |
| --- | --- |
| 1 | Developing Countries/ |
| 2 | (Africa or Caribbean or West Indies or South America or Latin America or Central America).hw,kf,ti,ab,cp. |
| 3 | (Benin or Burkina Faso or Burkina Fasso or Upper Volta or Burundi or Urundi or Central African Republic or Chad or Comoros or Comoro Islands or Comores or Mayotte or Congo or Democratic Republic of Congo or Republic of Zaire or Zaire or Eritrea or Ethiopia or Gambia or The Gambia or Guinea or Guinea Bissau or Liberia or Madagascar or Malagasy Republic or Malawi or Nyasaland or Mali or Mozambique or Niger or Rwanda or Ruanda or Sierra Leone or South Sudan or Tanzania or Togo or Togolese or Togolese Republic or Uganda or Zimbabwe or Cape Verde or Cabo Verde or Cameroon or Cameron or Camerons or Cote d'Ivoire or Ivory Coast or Ghana or Guiana or Guyana or Gold coast or Kenya or Lesotho or Basutoland or Mauritania or Nigeria or Sao Tome or Principe or Senegal or Swaziland or Zambia or Zimbabwe or Rhodesia or Cambodia or Khmer Republic or Kampuchea or Republic of Korea or North Korea or Korea or Kiribati or Laos or Lao or Lao Democratic Peoples Republic or Lao PDR or Federated States of Micronesia or Micronesia or Papua New Guinea or Philippines or Phillipines or Philipines or Samoa or Samoan Islands or Solomon Islands or Vanuatu or New Hebrides or Vietnam or Viet nam or Afghanistan or Somalia or Djibouti or French Somaliland or Egypt or United Arab Republic or Morocco or Ifni or Sudan or Syria or Syrian Arab Republic or West Bank and Gaza or Palestine or Yemen or Republic of Yemen or Bolivia or Guatemala or Guyana or Guiana or Honduras or Nicaragua or Haiti or El Salvador or Nepal or Bangladesh or Bhutan or India or Indonesia or Myanmar or Burma or Myanma or Pakistan or Sri Lanka or Timor leste or East Timor or East Timur or Armenia or Armenian or Georgia or Georgian Republic or Georgia Republic or Kosovo or Kyrgyz Republic or Kyrgyzstan or Kirghizia or Kyrgyz Republic or Kirghiz or Kirgizstan or Moldova or Moldovia or Tajikistan or Tadzhikistan or Tadjikistan or Tadzhik or Ukraine or Uzbekistan or Uzbek or Middle East).hw,kf,ti,ab,cp. |
| 4 | ((developing or less* developed or under developed or underdeveloped or middle income or low* income or underserved or under served or deprived or poor*) adj (countr* or nation? or state? or population? or world)).ti,ab. |
| 5 | ((developing or less* developed or under developed or underdeveloped or middle income or low* income) adj (economy or economies)).ti,ab. |
| 6 | (low* adj (gdp or gnp or gross domestic or gross national)).ti,ab. |
| 7 | (low adj3 middle adj3 countr*).ti,ab. |
| 8 | (lmic or lmics or third world or lami countr*).ti,ab. |
| 9 | transitional countr*.ti,ab. |
| 10 | 1 or 2 or 3 or 4 or 5 or 6 or 7 or 8 or 9 |
| 11 | exp Alcohol Drinking/ or exp alcoholic beverages/ |
| 12 | Drinking Behavior/ |
| 13 | (alcohol* adj5 (drink* or factor* or pattern* or habit* or consum* or unhealthy)).ti,ab. |
| 14 | (drink* adj5 (factor* or pattern* or habit* or consum* or unhealthy or bing*)).ti,ab. |
| 15 | (alcohol* or drink*).ti. |
| 16 | "tobacco use"/ or smoking/ |
| 17 | (tobacco or smoking or smoke or smoker?).ti,ab. |
| 18 | food habits/ or food preferences/ |
| 19 | diet, high-fat/ or portion size/ or serving size/ |
| 20 | exp Obesity/ |
| 21 | *Diet/ |
| 22 | exp carbonated beverages/ or energy drinks/ or *food/ or candy/ or exp *dietary fats/ or fast foods/ or fruit/ or exp vegetables/ |
| 23 | ((food or eating or diet*) adj5 (factor* or pattern* or habit* or consum* or unhealthy or healthy or healthful)).ti,ab. |
| 24 | ((high* or low*) adj2 (fat? or salt? or sugar or carbohydrate?)).ti,ab. |
| 25 | ((fat? or salt? or sodium or sugar? or carbohydrate?) adj5 (factor? or pattern? or habit? or consum* or eat? or eating)).ti,ab. |
| 26 | ((carbonated or sugar* or fizzy) adj2 (drink* or beverage?)).ti,ab. |
| 27 | (((unhealthy or healthy or healthful) adj3 (fat? or oil?)) or transfat? or trans fat?).ti,ab. |
| 28 | ((fruit? or vegetable? or fibre or fiber) adj5 (factor? or pattern? or habit? or consum* or eat? or eating)).ti,ab. |
| 29 | (junk food? or junkfood? or fast food? or fastfood? or snack*).ti,ab. |
| 30 | Motor Activity/ |
| 31 | exp *Exercise/ |
| 32 | *life style/ or sedentary lifestyle/ |
| 33 | (physical* adj3 (fit* or activ* or inactiv*)).ti,ab. |
| 34 | inactivity.ti,ab. |
| 35 | (sedentary adj3 (lifestyle* or life style* or behavio*)).ti,ab. |
| 36 | exercise*.ti. |
| 37 | or/ 36- 61 |
| 38 | ((poverty or impoverish* or inequalit* or inequit*) adj3 (reduc* or alleviat* or lower*)).ti,ab. |
| 39 | Taxes/ and ("Tobacco Use"/ or exp "Tobacco Use Cessation"/ or drinking behavior/ or exp alcohol drinking/ or exp Diet/ or food/ or exp dietary fats/ or fast foods/ or fruit/ or vegetables/ or Sodium Chloride, Dietary/ or Beverages/ or exp Exercise/ or Motor Activity/ or health behavior/ or risk reduction behavior/) |
| 40 | (tax or taxes or taxing or taxation).ti. |
| 41 | ((food? or diet* or vegetable? or fruit? or sugar* or fat or fats or sucrose or candy or sweet* or snack* or fastfood? or junkfood?) and (tax or taxes or taxing or taxation)).ti,ab. |
| 42 | ((beverage? or soda? or soft drink? or carbonated drink? or sugar sweetened drink?) and (tax or taxes or taxing or taxation)).ti,ab. |
| 43 | ((smok* or tobacco) and (tax or taxes or taxing or taxation)).ti,ab. |
| 44 | ((alcohol or drinking) and (tax or taxes or taxing or taxation)).ti,ab. |
| 45 | (subsidy or subsidies or incentiv* or voucher?).ti. |
| 46 | ((food? or diet* or vegetable? or fruit? or sugar* or fat or fats or sucrose or candy or sweet* or snack* or fastfood? or junkfood?) and (subsidy or subsidies or incentiv* or voucher?)).ti,ab. |
| 47 | ((beverage? or soda? or soft drink? or carbonated drink? or sugar sweetened drink?) and (subsidy or subsidies or incentiv* or voucher?)).ti,ab. |
| 48 | ((smok* or tobacco) and (subsidy or subsidies or incentiv* or voucher?)).ti,ab. |
| 49 | ((alcohol or drinking) and (subsidy or subsidies or incentiv* or voucher?)).ti,ab. |
| 50 | Economic Development/ |
| 51 | financial management/ or financial support/ or financing, organized/ or financing, personal/ |
| 52 | social change/ or social justice/ or feminism/ or womens rights/ or social support |
| 53 | socioeconomic factors/ or social capital/ or social environment |
| 54 | commerce/ or entrepreneurship/ or public-private sector partnerships/ or small business/ |
| 55 | ((economic* or business or commercial or financ* or income or livelihood?) adj5 (development or support* or intervention? or initiative?)).ti,ab. |
| 56 | ((employment or job? or labour market or labor market) adj5 (development or support* or intervention? or initiative?)).ti,ab. |
| 57 | (microfinanc* or micro-financ* or microcredit* or micro-credit*).ti,ab. |
| 58 | exp Social Planning/ |
| 59 | exp Fisheries/ |
| 60 | Health Promotion/ or Consumer Participation/ or Community Health Services/ or Community Health Planning/ |
| 61 | environmental policy/ |
| 62 | ((social or city or cities or town or urban or environment*) adj2 (plan or plans or planning or design or renew* or regenerat* or development)).ti,ab. |
| 63 | (communit* adj2 (regenerat* or renew* or mobili?ation or engagement or participation or involvement)).ti,ab. |
| 64 | agriculture/ or agricultural irrigation/ |
| 65 | Water Supply/ |
| 66 | exp Sanitary Engineering/ |
| 67 | ((agricultur* or farm*) adj5 (development or support or intervention? or initiative?)).ti,ab. |
| 68 | ((agricultur* or farm*) and (subsidy or subsidies or incentiv* or voucher?)).ti,ab. |
| 69 | ((water or sanitary or sanitation) adj5 (development or support or intervention? or initiative?)).ti,ab. |
| 70 | ((water or sanitary or sanitation) and (subsidy or subsidies or incentiv* or voucher?)).ti,ab. |
| 71 | irrigat*.ti,ab. |
| 72 | (Social Control, Formal/ or Legislation/) and ("Tobacco Use"/ or exp "Tobacco Use Cessation"/ or Tobacco Industry/ or Tobacco Smoke Pollution/ or drinking behavior/ or exp alcohol drinking/ or exp Diet/ or food/ or exp dietary fats/ or fast foods/ or fruit/ or vegetables/ or Sodium Chloride, Dietary/ or Beverages/ or exp Exercise/ or Motor Activity/ or health behavior/ or risk reduction behavior/) |
| 73 | smoke-free policy/ |
| 74 | exp Nutrition Policy/ |
| 75 | ((smoke or smoking or tobacco) adj2 (ban or bans or banned or free)).ti,ab. |
| 76 | ((smoke or smoking or tobacco) and (legislat* or law? or regulation or regulatory)).ti,ab. |
| 77 | ((food? or diet* or vegetable? or fruit? or sugar* or fat or fats or sucrose or candy or sweet* or snack* or fastfood? or junkfood?) and (legislat* or law? or regulation or regulatory)).ti,ab. |
| 78 | ((beverage? or soda? or soft drink? or carbonated drink? or sugar sweetened drink?) and (legislat* or law? or regulation or regulatory)).ti,ab. |
| 79 | ((alcohol or drinking) and (legislat* or law? or regulation or regulatory)).ti,ab. |
| 80 | Social Marketing/ |
| 81 | (Marketing/ or advertising as topic/ or Mass Media/ or product packaging/ or product labeling/) and ("Tobacco Use"/ or exp "Tobacco Use Cessation"/ or drinking behavior/ or exp alcohol drinking/ or exp Diet/ or food/ or exp dietary fats/ or fast foods/ or fruit/ or vegetables/ or Sodium Chloride, Dietary/ or Beverages/ or exp Exercise/ or Motor Activity/ or health behavior/ or risk reduction behavior/) |
| 82 | food packaging/ or food labeling/ |
| 83 | ((food? or diet* or vegetable? or fruit? or sugar* or fat or fats or sucrose or candy or sweet* or snack* or fastfood? or junkfood?) and (marketing or adverti?ing or sponsorship? or label* or pack*)).ti,ab. |
| 84 | ((beverage? or soda? or soft drink? or carbonated drink? or sugar sweetened drink?) and (marketing or adverti?ing or sponsorship? or label* or pack*)).ti,ab. |
| 85 | ((smok* or tobacco) and (marketing or adverti?ing or sponsorship? or label* or pack*)).ti,ab. |
| 86 | ((alcohol or drinking) and (marketing or adverti?ing or sponsorship? or label* or pack*)).ti,ab. |
| 87 | (salt adj3 (intake or reduc* or lower*)).ti,ab. |
| 88 | Health Promotion/ and (Neoplasms/ or Obesity/ or Diabetes Mellitus, Type 2/ or Smoking/ or Risk Factors/ or Cardiovascular Diseases/ or Hypertension/) |
| 89 | Patient Education as Topic/ or Counseling/ or Patient Compliance/ or Motivational Interviewing/ and (Neoplasms/ or Obesity/ or Diabetes Mellitus, Type 2/ or Smoking/ or Risk Factors/ or Cardiovascular Diseases/ or Hypertension/) |
| 90 | Health Education/ or Health Knowledge, Attitudes, Practice/ and (Life Style/ or Food Habits/ or Diet/ or Health Behavior/ or Obesity/ or Motor Activity/ |
| 91 | exp Mouth Neoplasms/mo, pc [Mortality, Prevention & Control] |
| 92 | exp Areca/ |
| 93 | Food legislation/ and (dietary fats/ or sodium/ or carbohydrates/) |
| 94 | Food supply/ and (dietary fats/ or sodium/ or carbohydrates/) |
| 95 | best Buys.mp |
| 96 | or/63-120 |
| 97 | 10 and 37 and 96 |
| 98 | trials/ or exp clinical trials/ or feasibility studies/ or intervention/ |
| 99 | (intervention* or pilot or feasability or trial).ti. |
| 100 | program development/ or program effectiveness/ or program evaluation/ or programs/ |
| 101 | (program* or model*).ti. |
| 102 | (program* adj2 (evaluat* or monitor* or implement* or develop*)).ti,ab. |
| 103 | ((econ* or cost* or socioecon*) adj3 model*).ti,ab. |
| 104 | 123 or 124 or 125 or 126 or 127 or 128 |
| 105 | 122 and 129 |

Search Terms and Strategy (example taken from MEDLINE search for NCD morbidity and mortality):

| # | Searches |
| --- | --- |
| 1 | Developing Countries/ |
| 2 | (Africa or Caribbean or West Indies or South America or Latin America or Central America).hw,kf,ti,ab,cp. |
| 3 | (Benin or Burkina Faso or Burkina Fasso or Upper Volta or Burundi or Urundi or Central African Republic or Chad or Comoros or Comoro Islands or Comores or Mayotte or Congo or Democratic Republic of Congo or Republic of Zaire or Zaire or Eritrea or Ethiopia or Gambia or The Gambia or Guinea or Guinea Bissau or Liberia or Madagascar or Malagasy Republic or Malawi or Nyasaland or Mali or Mozambique or Niger or Rwanda or Ruanda or Sierra Leone or South Sudan or Tanzania or Togo or Togolese or Togolese Republic or Uganda or Zimbabwe or Cape Verde or Cabo Verde or Cameroon or Cameron or Camerons or Cote d'Ivoire or Ivory Coast or Ghana or Guiana or Guyana or Gold coast or Kenya or Lesotho or Basutoland or Mauritania or Nigeria or Sao Tome or Principe or Senegal or Swaziland or Zambia or Zimbabwe or Rhodesia or Cambodia or Khmer Republic or Kampuchea or Republic of Korea or North Korea or Korea or Kiribati or Laos or Lao or Lao Democratic Peoples Republic or Lao PDR or Federated States of Micronesia or Micronesia or Papua New Guinea or Philippines or Phillipines or Philipines or Samoa or Samoan Islands or Solomon Islands or Vanuatu or New Hebrides or Vietnam or Viet nam or Afghanistan or Somalia or Djibouti or French Somaliland or Egypt or United Arab Republic or Morocco or Ifni or Sudan or Syria or Syrian Arab Republic or West Bank and Gaza or Palestine or Yemen or Republic of Yemen or Bolivia or Guatemala or Guyana or Guiana or Honduras or Nicaragua or Haiti or El Salvador or Nepal or Bangladesh or Bhutan or India or Indonesia or Myanmar or Burma or Myanma or Pakistan or Sri Lanka or Timor leste or East Timor or East Timur or Armenia or Armenian or Georgia or Georgian Republic or Georgia Republic or Kosovo or Kyrgyz Republic or Kyrgyzstan or Kirghizia or Kyrgyz Republic or Kirghiz or Kirgizstan or Moldova or Moldovia or Tajikistan or Tadzhikistan or Tadjikistan or Tadzhik or Ukraine or Uzbekistan or Uzbek or Middle East).hw,kf,ti,ab,cp. |
| 4 | ((developing or less* developed or under developed or underdeveloped or middle income or low* income or underserved or under served or deprived or poor*) adj (countr* or nation? or state? or population? or world)).ti,ab. |
| 5 | ((developing or less* developed or under developed or underdeveloped or middle income or low* income) adj (economy or economies)).ti,ab. |
| 6 | (low* adj (gdp or gnp or gross domestic or gross national)).ti,ab. |
| 7 | (low adj3 middle adj3 countr*).ti,ab. |
| 8 | (lmic or lmics or third world or lami countr*).ti,ab. |
| 9 | transitional countr*.ti,ab. |
| 10 | 1 or 2 or 3 or 4 or 5 or 6 or 7 or 8 or 9 |
| 11 | cardiovascular diseases/ or heart diseases/ or vascular diseases/ or cerebrovascular diseases/ |
| 12 | exp Myocardial Ischemia/ |
| 13 | Heart Failure/ |
| 14 | exp brain ischemia/ or exp stroke/ |
| 15 | exp Diabetes Mellitus, Type 2/ |
| 16 | lung diseases, obstructive/ or exp pulmonary disease, chronic obstructive/ |
| 17 | exp *Neoplasms/ |
| 18 | ((cardiovascular or cardio-vascular) adj3 disease*).ti,ab. |
| 19 | ((cardiovascular or cardio-vascular) adj3 (event* or outcome* or risk*)).ti,ab. |
| 20 | ((coronary or heart or myocard*) adj3 disease*).ti,ab. |
| 21 | ((coronary or heart or myocard*) adj3 (event* or outcome* or risk*)).ti,ab. |
| 22 | ((ischaemic or ischemic or ischaemia or ischemia) adj3 disease*).ti,ab. |
| 23 | ((ischaemic or ischemic or ischaemia or ischemia) adj3 (event* or outcome* or risk*)).ti,ab. |
| 24 | myocardial infarct*.ti,ab. |
| 25 | ((cerebrovascular or vascular) adj3 disease*).ti,ab. |
| 26 | ((cerebrovascular or vascular) adj3 (event* or outcome* or risk*)).ti,ab. |
| 27 | stroke.ti,ab. |
| 28 | heart failure.ti,ab. |
| 29 | diabet*.ti. |
| 30 | ((type 2 or type ii or noninsulin dependent or non insulin dependent or adult onset or maturity onset or obes*) adj2 diabet*).ti,ab. |
| 31 | (niddm or t2dm or tiidm).ti,ab. |
| 32 | (chronic adj2 (lung or pulmonary)).ti,ab. |
| 33 | chronic obstructive pulmonary disease.ti,ab. |
| 34 | (neoplas* or cancer* or carcinoma* or tumor* or tumour* or malignan* or leukaemia or leukemia or lymphoma?).ti,ab. |
| 35 | 11 or 12 or 13 or 14 or 15 or 16 or 17 or 18 or 19 or 20 or 21 or 22 or 23 or 24 or 25 or 26 or 27 or 28 or 29 or 30 or 31 or 32 or 33 or 34 |
| 36 | ((poverty or impoverish* or inequalit* or inequit*) adj3 (reduc* or alleviat* or lower*)).ti,ab. |
| 37 | Taxes/ and ("Tobacco Use"/ or exp "Tobacco Use Cessation"/ or drinking behavior/ or exp alcohol drinking/ or exp Diet/ or food/ or exp dietary fats/ or fast foods/ or fruit/ or vegetables/ or Sodium Chloride, Dietary/ or Beverages/ or exp Exercise/ or Motor Activity/ or health behavior/ or risk reduction behavior/) |
| 38 | (tax or taxes or taxing or taxation).ti. |
| 39 | ((food? or diet* or vegetable? or fruit? or sugar* or fat or fats or sucrose or candy or sweet* or snack* or fastfood? or junkfood?) and (tax or taxes or taxing or taxation)).ti,ab. |
| 40 | ((beverage? or soda? or soft drink? or carbonated drink? or sugar sweetened drink?) and (tax or taxes or taxing or taxation)).ti,ab. |
| 41 | ((smok* or tobacco) and (tax or taxes or taxing or taxation)).ti,ab. |
| 42 | ((alcohol or drinking) and (tax or taxes or taxing or taxation)).ti,ab. |
| 43 | (subsidy or subsidies or incentiv* or voucher?).ti. |
| 44 | ((food? or diet* or vegetable? or fruit? or sugar* or fat or fats or sucrose or candy or sweet* or snack* or fastfood? or junkfood?) and (subsidy or subsidies or incentiv* or voucher?)).ti,ab. |
| 45 | ((beverage? or soda? or soft drink? or carbonated drink? or sugar sweetened drink?) and (subsidy or subsidies or incentiv* or voucher?)).ti,ab. |
| 46 | ((smok* or tobacco) and (subsidy or subsidies or incentiv* or voucher?)).ti,ab. |
| 47 | ((alcohol or drinking) and (subsidy or subsidies or incentiv* or voucher?)).ti,ab. |
| 48 | Economic Development/ |
| 49 | financial management/ or financial support/ or financing, organized/ or financing, personal/ |
| 50 | social change/ or social justice/ or feminism/ or womens rights/ or social support |
| 51 | socioeconomic factors/ or social capital/ or social environment |
| 52 | commerce/ or entrepreneurship/ or public-private sector partnerships/ or small business/ |
| 53 | ((economic* or business or commercial or financ* or income or livelihood?) adj5 (development or support* or intervention? or initiative?)).ti,ab. |
| 54 | ((employment or job? or labour market or labor market) adj5 (development or support* or intervention? or initiative?)).ti,ab. |
| 55 | (microfinanc* or micro-financ* or microcredit* or micro-credit*).ti,ab. |
| 56 | exp Social Planning/ |
| 57 | exp Fisheries/ |
| 58 | Health Promotion/ or Consumer Participation/ or Community Health Services/ or Community Health Planning/ |
| 59 | environmental policy/ |
| 60 | ((social or city or cities or town or urban or environment*) adj2 (plan or plans or planning or design or renew* or regenerat* or development)).ti,ab. |
| 61 | (communit* adj2 (regenerat* or renew* or mobili?ation or engagement or participation or involvement)).ti,ab. |
| 62 | agriculture/ or agricultural irrigation/ |
| 63 | Water Supply/ |
| 64 | exp Sanitary Engineering/ |
| 65 | ((agricultur* or farm*) adj5 (development or support or intervention? or initiative?)).ti,ab. |
| 66 | ((agricultur* or farm*) and (subsidy or subsidies or incentiv* or voucher?)).ti,ab. |
| 67 | ((water or sanitary or sanitation) adj5 (development or support or intervention? or initiative?)).ti,ab. |
| 68 | ((water or sanitary or sanitation) and (subsidy or subsidies or incentiv* or voucher?)).ti,ab. |
| 69 | irrigat*.ti,ab. |
| 70 | (Social Control, Formal/ or Legislation/) and ("Tobacco Use"/ or exp "Tobacco Use Cessation"/ or Tobacco Industry/ or Tobacco Smoke Pollution/ or drinking behavior/ or exp alcohol drinking/ or exp Diet/ or food/ or exp dietary fats/ or fast foods/ or fruit/ or vegetables/ or Sodium Chloride, Dietary/ or Beverages/ or exp Exercise/ or Motor Activity/ or health behavior/ or risk reduction behavior/) |
| 71 | smoke-free policy/ |
| 72 | exp Nutrition Policy/ |
| 73 | ((smoke or smoking or tobacco) adj2 (ban or bans or banned or free)).ti,ab. |
| 74 | ((smoke or smoking or tobacco) and (legislat* or law? or regulation or regulatory)).ti,ab. |
| 75 | ((food? or diet* or vegetable? or fruit? or sugar* or fat or fats or sucrose or candy or sweet* or snack* or fastfood? or junkfood?) and (legislat* or law? or regulation or regulatory)).ti,ab. |
| 76 | ((beverage? or soda? or soft drink? or carbonated drink? or sugar sweetened drink?) and (legislat* or law? or regulation or regulatory)).ti,ab. |
| 77 | ((alcohol or drinking) and (legislat* or law? or regulation or regulatory)).ti,ab. |
| 78 | Social Marketing/ |
| 79 | (Marketing/ or advertising as topic/ or Mass Media/ or product packaging/ or product labeling/) and ("Tobacco Use"/ or exp "Tobacco Use Cessation"/ or drinking behavior/ or exp alcohol drinking/ or exp Diet/ or food/ or exp dietary fats/ or fast foods/ or fruit/ or vegetables/ or Sodium Chloride, Dietary/ or Beverages/ or exp Exercise/ or Motor Activity/ or health behavior/ or risk reduction behavior/) |
| 80 | food packaging/ or food labeling/ |
| 81 | ((food? or diet* or vegetable? or fruit? or sugar* or fat or fats or sucrose or candy or sweet* or snack* or fastfood? or junkfood?) and (marketing or adverti?ing or sponsorship? or label* or pack*)).ti,ab. |
| 82 | ((beverage? or soda? or soft drink? or carbonated drink? or sugar sweetened drink?) and (marketing or adverti?ing or sponsorship? or label* or pack*)).ti,ab. |
| 83 | ((smok* or tobacco) and (marketing or adverti?ing or sponsorship? or label* or pack*)).ti,ab. |
| 84 | ((alcohol or drinking) and (marketing or adverti?ing or sponsorship? or label* or pack*)).ti,ab. |
| 85 | (salt adj3 (intake or reduc* or lower*)).ti,ab. |
| 86 | Health Promotion/ and (Neoplasms/ or Obesity/ or Diabetes Mellitus, Type 2/ or Smoking/ or Risk Factors/ or Cardiovascular Diseases/ or Hypertension/) |
| 87 | Drug therapy, combination and (Diabetes Mellitus, Type 2/ or Cardiovascular Diseases/ or Hypertension/) |
| 88 | Patient Education as Topic/ or Counseling/ or Patient Compliance/ or Motivational Interviewing/ and (Neoplasms/ or Obesity/ or Diabetes Mellitus, Type 2/ or Smoking/ or Risk Factors/ or Cardiovascular Diseases/ or Hypertension/) |
| 89 | Health Education/ or Health Knowledge, Attitudes, Practice/ and (Life Style/ or Food Habits/ or Diet/ or Health Behavior/ or Obesity/ or Motor Activity/ |
| 90 | Air Pollution, Indoor/ and Pulmonary Disease, Chronic Obstructive/ |
| 91 | exp Mouth Neoplasms/mo, pc [Mortality, Prevention & Control] |
| 92 | exp Areca/ |
| 93 | Hydroxymethylglutaryl-CoA Reductase Inhibitors/ or Simvastatin/ or Aspirin/ or Metformin/ or  Adrenergic beta-Antagonists/ or Propranolol/ |
| 94 | Food legislation/ and (dietary fats/ or sodium/ or carbohydrates/) |
| 95 | Food supply/ and (dietary fats/ or sodium/ or carbohydrates/) |
| 96 | Drug Therapy, Combination/ adj (Diabetes Mellitus, Type 2/ or Cardiovascular Diseases/) |
| 97 | Papanicolaou Test/ |
| 98 | Mass Screening/ and (Uterine Cervical Neoplasms/ or Cervical Intraepithelial Neoplasia/) |
| 99 | ((cervical or pap) adj3 screen*).ti,ab. |
| 100 | Hepatitis B Vaccines/ |
| 101 | ((hepatitis b or hep b) adj3 (vaccin* or immuni?ation or immuni?e)).ti,ab. |
| 102 | *Aspirin/ |
| 103 | Primary Prevention/ and Aspirin/ |
| 104 | (aspirin or acetylsalicylic acid).ti,ab. |
| 105 | best Buys.mp |
| 106 | or/36-105 |
| 107 | 10 and 35 and 106 |
| 108 | limit 107 to "reviews (maximizes specificity)" |
| 109 | limit 91 to "therapy (maximizes sensitivity)" |
| 110 | feasibility studies/ or intervention studies/ or pilot projects/ |
| 111 | (intervention* or pilot or feasability).ti. |
| 112 | evaluation studies as topic/ or program evaluation/ |
| 113 | (program* or model*).ti. |
| 114 | (program* adj2 (evaluat* or monitor* or implement* or develop*)).ti,ab. |
| 115 | ((econ* or cost* or socioecon*) adj3 model*).ti,ab. |
| 116 | 111 or 112 or 113 or 114 or 115 or 116 |
| 117 | 107 and 117 |
| 118 | 108 or 109 or 117 |
