# Supplementary material for: The impact of poverty reduction and development interventions on non-communicable diseases and their behavioural risk factors in low and lower-middle income countries: A systematic review
Source: PLoS One. 2018 Feb 23;13(2):e0193378. doi: 10.1371/journal.pone.0193378 (PMC5825092; doi:10.1371/journal.pone.0193378)
Supplement: S3 Table — Measurement and outcome criteria for review inclusion. (DOCX) [file pone.0193378.s003.docx]

S3 Table. Eligibility Criteria

Based on a review of work by international development agencies including UN agencies, bi/multi-lateral agencies and non-government organisations the list of interventions below was produced. The included list aimed to ensure interventions targeting absolute and relative poverty were included.

| **Included Development Intervention Themes** | **Included NCD morbidity and mortality outcomes [8]** | **Included NCD behavioural risk factor outcomes [8]** |
| --- | --- | --- |
| - Economic development interventions (including financial management/ support, small business promotion, entrepreneurship, market linkage) - Social Change interventions (including projects which support gender equality, social justice, social support) - Social protection interventions (including conditional and unconditional cash transfer, food for work, government subsidies, government taxes, microcredit programmes) - Employment programs (training, access, loans) - Fisheries programmes - Environmental protection programmes - Agricultural programmes - Water and sanitisation programmes | - Cardiovascular disease (including myocardial infarction, heart failure, brain ischemia, stroke, heart disease, coronary artery disease, cerebrovascular events, vascular events, heart failure) - Diabetes (including type 2 diabetes, non-insulin dependent diabetes, type 1 diabetes, insulin resistance, impaired glucose tolerance) - Cancer (including neoplasms, carcinoma, tumors, malignancy, leukaemia, lymphoma) - Chronic respiratory diseases (including chronic obstructive pulmonary disease, chronic lung/pulmonary conditions, asthma, lung diseases) | - Tobacco use (tobacco smoking or chewing, tobacco control policies) - Physical inactivity (interventions which impacted on physical activity levels) - Unhealthy diet (interventions which influence diet composition, fruit, vegetable, salt, fat, sugar intake) - Harmful use of alcohol (interventions which impacted on alcohol consumption)   Interventions which impacted on obesity (via diet or physical inactivity) were also considered within the review |
