# Supplementary material for: The impact of poverty reduction and development interventions on non-communicable diseases and their behavioural risk factors in low and lower-middle income countries: A systematic review
Source: PLoS One. 2018 Feb 23;13(2):e0193378. doi: 10.1371/journal.pone.0193378 (PMC5825092; doi:10.1371/journal.pone.0193378)
Supplement: S4 Table — Quality assessment of included papers. (DOCX) [file pone.0193378.s004.docx]

S4 Table. Quality and Bias Assessment of Studies

Cochrane Risk of Bias Tool Assessment for Randomised Controlled Trials

| Author, date, country [ref]  Theme | Domain | | | | | | |
| --- | --- | --- | --- | --- | --- | --- | --- |
|  | Sequence Generation | Allocation Concealment | Blinding of Participants and Researchers | Blinding of Outcome Assessment | Incomplete Outcome Data | Selective Outcome Reporting Bias | Other Bias |
| **Hotz C, 2012, Mozambique [19]**  Bio-fortification | **Unclear**  Insufficient information- randomisation process not described. Convenience sampling used within randomised participants. | **Unclear**  insufficient information -not described | **Unclear**  Insufficient information on blinding. Participant clusters kept 5km apart. | **Unclear**  Insufficient information on blinding | **High**  9-11% participants lost to follow up with no reason provided | **Low**  All stated outcomes reported on | **Unclear**  Baseline taken outside of harvest season, follow-up inside season. No follow-up reports of height/weight or physiological outcomes. No discussion of limitations |
| **Hotz C, 2012, Uganda [20]**  Bio-fortification | **Unclear**  insufficient information- randomisation process not described. Convenience sampling used within randomised participants. | **Unclear**  insufficient information -not described | **Unclear**  Insufficient information on blinding. Participant clusters kept 5km apart. | **Unclear**  Insufficient information on blinding | **High**  Between 2-15% participants lost to follow up, description not provided and participants were replaced | **Unclear**  Designed to only assess the impact on participants with low serum retinol | **Low**  Accounted for changes in food sources of vitamin A |
| **Weiser SD, 2015, Kenya [24]**  Social Protection Program | **Low**  Independent biostatistician used computer random number generator | **Low**  Geographically separated health clinics | **High** Researchers and participants not blinded | **High**  Not blinded | **Low**  92-97% follow-up obtained | **Low**  All intended outcomes measured and reported on | **High**  Cluster level variables not accounted for and significant differences in baseline characteristics |
| **Olney DK, 2015, Burkina Faso [28]**  **Agricultural Diversification** | **Unclear**  Randomisation not described | **Unclear**  Not described | **Unclear**  Not described | **Unclear**  Not described | **Low**  Attrition explained and controlled for | **Unclear**  Intervention involved women but outcomes not measured | **Unclear** No reporting on malaria and zinc status despite endemic. Discussed in limitations. |

**Quality Assessment of observational studies using NOS**

| **Study** | **Selection** | | | | **Comparability** | **Outcome** | | |
| --- | --- | --- | --- | --- | --- | --- | --- | --- |
| **Author, Date, Country [ref]** | **Representativeness of exposed cohort** | **Selection of the non-exposed cohort** | **Ascertainment of exposure** | **Demonstration that outcome of interest was not present at start of study** | **Comparability based on design and analysis** | **Assessment of outcome** | **Duration of follow-up** | **Adequacy of follow-up** |
| **Morris SS, 1999, Honduras [15]**  **Social Protection Programme** | B* | A* | B* | B | - | B* | A* | D |
|  | Convenient sampling | Matched households | Structured interview | Not described | No adjustments noted | Record linkage | 2 years | No statement |
| **Kishtwaria J, 2012, India [17]**  **Physical Activity** | A* | C | A* | A* | - | B* | A* | A* |
|  | Convenient sampling of target population | No controls | Secure report of results | Baseline measures | No description | Record linkage | Yes | All participants |
| **Low J, 2007, Mozambique^18^**  **Biofortification** | B* | A* | A* | A* | A** | B* | A* | B* |
|  | Convenient sampling of high needs population | Randomly assigned from same population | Structured interview + anthropometry and blood samples | Baseline data | Controlled for infection, household differences and income | Record linkage | 2 years | 90% retained, no description of those lost |
| **Kidala D, 2000, Tanzania [21]**  **Biofortification** | B* | B | A* | B | B* | B* | A* | B* |
|  | All households in study community | Different population | Blood and stool sample | Only whole population baseline data – not participant | Controlled for infection, vitamin A supplementation but not baseline differences | Record linkage | 5 years | Excluded infection but used in comparison measures |
| **Marquis GS, 2015, Ghana [23]**  **Social Protection Program** | B* | A* | A* | B | B* | B* | A* | C |
|  | Convenient sampling of female caregivers and children aged 2-5 | Children from matched communities | Anthropometry | Baseline statistics show discrepancies with income, ASF, Age, WAZ and BAZ | Controlled for factors association with anthropometry measures, not infection | Record linkage | 16 months | 69% intervention, 83% control lost in follow up providing insufficient to power to detect changes. Inaccurate age assessments meant large SDs |
| **Langendorf, 2014, Niger^22^**  **Social Protection Program** | B* | C | A* | A* | B* | B* | B | B* |
|  | Convenience sample of children | No comparison group | Anthropometry | Baseline statistics | Controlled for group differences | Record linkage | Only 4 months | Max of 16% lost to follow-up, explained |
| **Mascie-Taylor CGN, 2010, Bangladesh [26]** | B* | A* | B* | A* | B* | B8 | A* | B* |
|  | Convenient sampling | Matched households | Structured interview, anthropometry | Baseline measures | Adjusted for age and gender in children | Record linkage | 10 weeks- study completion, would need longer to check sustainability | 11.6% lost to follow up- some removed due to identification of severe underweight |
| **Hanson M, 2011, Federated States of Micronesia [29]**  **Agricultural- diversification** | B* | B | B* | B | - | C | A* | D |
|  | Convenient sample | Different population | FFQ with interview | Baseline taken from different population | No comparison group | Self reported FFQ and opinion | 1 year | No statement |
| **Kariuki LW, 2011, Kenya [30]**  **Agricultural diversification** | B* | B | B | A* | - | C | A* | D |
|  | Convenient sample of women’s groups | Only compared between intervention groups | Self-reported, not described | Baseline measures | No adjustments | Self-report | Programme completion | No statement |
| **Bamji MS, 2011, India [31]**  **Agricultural diversification** | C | C | B* | A* | - | B* | A* | C |
|  | Convenient sample of farmers attending workshop | No description | Structured interview | Baseline data | No description | Record linkage | 3 years | No description and added extra participants |
| **Singh H, 2014, India [32]**  **Agricultural diversification** | B* | C | B* | A* | - | B* | A* | D |
|  | Convenience sampling | No control | Structured interview | Baseline measures | None described | Record linkage | Years | Not mentioned |
| **Jones KM, 2005, Nepal [34]**  **Agricultural diversification** | B* | A* | B* | B | - | C | A* | D |
|  | Convenient sampling | Drawn from same community | Structured interview | Not described | No adjustments | Self- report | 36 months | No statement |
| **Ahmed MM, 2000, Ethiopia [35]**  **Livestock diversification** | B* | A* | B* | B | A* | B* | A* | D |
|  | Convenience sampling | Matched control group | Structured interview | No baseline | Controls for difference in cows but no other factors (e.g. land holding) | Record linkage | Approx. 5 years | No statement |
| **Walingo MK, 2012, Kenya [36]**  **Livestock diversification** | B* | A* | B* | B | - | B* | A* | D |
|  | Convenience sampling | Matched participants | Structured interview | No baseline | None described | Record linkage | At least 3 years | Convenient sample taken |
| **Ahmad KM, 2010, Bangladesh [37]**  **Livestock diversification** | B* | A* | B* | A* | - | B* | A* | D |
|  | Convenience sampling | Matched participants | Structured interview | Baseline measures | None described | Record linkage | 3 Years | Not mentioned |
| **Ekesa BN, 2013, Burundi, Democratic republic of Congo, Rwanda [40]**  **Multi-component** | B* | A* | C | B | B* | C | A* | D |
|  | Convenient sampling of farming families | Randomly assigned from same population | Self-report | No description | Controlled for some other influences | Self- report | 4 years | No statement |
| **Moench-Pfanner R, 2005, Indonesia [42]**  **Social Protection Program** | B* | B | B* | A* | B* | B* | A* | B* |
|  | Convenient sample of mothers from beneficiary households | Controls showed significantly different baseline characteristics | Structured interview, blood samples and qualitative | Baseline statistics | Controlled for baseline differences but not infection | Record linkage | 4-18 months | Lost population reported |

**Quality assessment of cross-sectional studies using modified version of NOS**

| **Author, year, reference, country, intervention style** | **Selection** | | | | **Comparability** | **Outcome** | |
| --- | --- | --- | --- | --- | --- | --- | --- |
|  | **Sample representativeness** | **Sample size** | **Non-respondents** | **Ascertainment of Exposure** | **Control for confounders** | **Outcome Assessment** | **Statistical test** |
| **Nair M, 2013, India [25]**  Social Protection programme | A* | A* | B* | A** | B* | B** | A* |
|  | Random sampling of intervention/control households | Justified | Response rate 89.6% | Anthropometry  FANTA-2, FAO dietary diversity score | Regression analysis controlled for SES, poverty and caste | Record linkage | Univariate logistic regression analyses, Chi-square, Comparative Fit Index and RMSEA |
| **Olney DK,2009, Cambodia [27]**  Agricultural diversification programme | B* | A* | C | A* | - | B** | A* |
|  | Convenient sampling by NGO | Justified | No description | Validated measures of anthropometry and diet | Seasonal differences in baseline/end line not controlled for | Record linkage | Chi-squared test |
| **Sharma KR, 1999, Nepal [33]**  Agricultural diversification | B* | A* | C | A** | B* | B** | A* |
|  | Somewhat representative, non-randomised | All households interviewed | No description | Anthropometry and questionnaire | Multi-variate regression analysis | Record linkages | ANOVA |
| **Remans R, 2011, Ethiopia, Ghana, Kenya, Malawi, Mali, Nigeria, Senegal, Tanzania, Uganda [40]**  **Multi-Component intervention** | B* | A* | C | A* | B* | B* | A* |
|  | Convenient sampling | Justified | No description, convenience sampling based on availability of results | Validated measure of anthropology, Food Diversity | Controlled for major confounders within communities but no comparison group | Record linkage | Appropriate measure |
| **Hop LT, 2003, Vietnam [41]**  Multi-component intervention | A* | A | B* | A* | - | B* | B |
|  | Whole population survey | Justified and satisfactory | No description but national data used | Validated measure | No description | Record linkage | Correlation only |
